# Machine learning predicts and provides insights into milk acidification rates of *Lactococcus lactis*

**DOI:** 10.1371/journal.pone.0246287

**Published:** 2021-03-15

**Authors:** Signe Tang Karlsen, Tammi Camilla Vesth, Gunnar Oregaard, Vera Kuzina Poulsen, Ole Lund, Gemma Henderson, Jacob Bælum

**Affiliations:** 1 Chr. Hansen A/S, Hoersholm, Denmark; 2 National Food Institute, Technical University of Denmark, Lyngby, Denmark; Technical Educational Institute of Peloponnese, GREECE

## Abstract

*Lactococcus lactis* strains are important components in industrial starter cultures for cheese manufacturing. They have many strain-dependent properties, which affect the final product. Here, we explored the use of machine learning to create systematic, high-throughput screening methods for these properties. Fast acidification of milk is such a strain-dependent property. To predict the maximum hourly acidification rate (V_max_), we trained Random Forest (RF) models on four different genomic representations: Presence/absence of gene families, counts of Pfam domains, the 8 nucleotide long subsequences of their DNA (8-mers), and the 9 nucleotide long subsequences of their DNA (9-mers). V_max_ was measured at different temperatures, volumes, and in the presence or absence of yeast extract. These conditions were added as features in each RF model. The four models were trained on 257 strains, and the correlation between the measured V_max_ and the predicted V_max_ was evaluated with Pearson Correlation Coefficients (PC) on a separate dataset of 85 strains. The models all had high PC scores: 0.83 (gene presence/absence model), 0.84 (Pfam domain model), 0.76 (8-mer model), and 0.85 (9-mer model). The models all based their predictions on relevant genetic features and showed consensus on systems for lactose metabolism, degradation of casein, and pH stress response. Each model also predicted a set of features not found by the other models.

## Introduction

It is important to understand the genomic basis for microbial traits in order to produce better microorganisms for food cultures, probiotics, and production of enzymes, medicine, and chemicals. While the arrival of whole-genome sequencing has revealed the DNA sequences of microorganisms, the functions of many microbial genes are as yet unknown. Furthermore, many phenotypes are complex and depend on the interactions of multiple genes. Phenotypes may therefore still be difficult to predict even with good knowledge of the genes directly involved and their individual functions. When little previous knowledge of the phenotype is available, machine learning provides a fast and cheap way to model the phenotype; simultaneously providing a valuable screening tool and insights into the mode of action. We here demonstrate how machine learning can be applied to genome data in order to predict complex traits and identify genes involved in causing them.

*Lactococcus lactis* is widely used in industrial starter cultures for cheese and butter-milk production. The choice of starter culture affects both the texture, flavour, and aroma of the cheese [1]. It is therefore valuable to be able to characterize large numbers of potential new starter culture strains in a fast and cheap manner. An important characteristic is the milk acidification activity of a strain. This can be measured in terms of decrease in pH over time as lactic acid is produced from lactose. To compare these measurements, acidification can be quantified by for instance maximum hourly acidification rate (V_max_), time to reach V_max_, and lowest pH obtained. These acidification kinetics of *L. lactis* are strain-dependent and cannot be inferred from species or subspecies. In addition to high-throughput screening assays [2], *in-silico* screening of strains based on whole-genome sequencing data is now commonly used for various types of strain characterization. *In-silico* screening scales well, enabling fast screening of many strains. However, while some bacterial traits can be inferred from the presence of a single gene (for instance many types of antibiotic resistance [3]), other traits are more complex and building genome-based screening tools for these is not a trivial task. Milk acidification in *L. lactis* is such a phenotype, which depends on multiple genes and interactions. Independently, many of the mechanisms involved are well-described in literature, but determining the results of their combined contributions is still imprecise. Machine learning has become state of the art for extracting essential information from complex data structures in fields such as image recognition, speech recognition, and text mining. Machine learning models are mathematical models such as linear regression, which are constructed directly from data. Based on a set of features and known target values, a model is constructed that can predict target values from feature values. In recent years, machine learning has also been applied for the prediction of different bacterial phenotypes. Traitar [4] is an example of such a tool, which was developed to predict a wide array of phenotypes such as the ability to grow aerobically, to form spores, or ferment glucose. Other studies focus on predicting resistance or susceptibility to various antibiotics [5–7] or minimum inhibitory concentrations [8]. While many prediction models focus on clinically relevant phenotypes for pathogenic bacteria, two studies have applied classification models specifically to *Lactococcus* strains [9, 10] predicting antibiotic resistance, metal resistance, and ability to grow on various carbon sources, on milk, and on polysaccharides.

The best way to represent genomes in a machine learning model depends on what causes the phenotype. Some bacterial traits can be predicted by the presence of certain combinations of genes. One genomic representation is thus the presence/absence of genes. One study thus used Ortholog Groups (OGs) to define the presence/absence of genes [9]. Another study used a pangenome to identify the presence/absence of genes [7]. In a pangenome, genes from different bacterial strains are grouped together based on sequence similarity. However, sometimes genes with lower sequence similarity have the same function. Another study used Pfam domain families to represent the genomes [4]. Pfam domains are functional or structural regions of the genes [11]. Using these allows a model to discriminate based on functional profiles rather than full-length protein sequence similarity. However, sometimes the difference in phenotype is expected to arise from point mutations or from non-coding areas of the genome. To identify these types of signals, genomes can be represented by k-mers. These are sub-sequences of the DNA of length k. This genome representation has also been used for creating phenotype prediction models [5, 12–14].

When predicting bacterial phenotypes with machine learning, the number of features is typically large compared to the number of target values and the data is very noisy in the sense that most of the features have no effect on the phenotype. The machine learning algorithm Random Forest (RF) [15] is well suited for this type of data. Other algorithms which have been used for bacterial phenotype prediction include Support Vector Machines [4], Neural Networks [6, 7], Gradient Boosted Decision Trees [7], XGBoost [8], Logistic Regression [6, 12, 13], and Linear Regression [12]. A RF is not prone to over-fitting when given many features and can handle non-linear associations and highly correlating features [16]. A RF consists of multiple decision trees, which are each built from a different subset of the training data. A decision tree makes a prediction based on a set of rules, which are learned from the data. When solving regression problems, the final prediction of the RF model is the average of the decision tree predictions, and when solving classification problems, the final prediction of the RF model is the most common of the decision tree predictions. A RF model is constructed by using a part of the data, the training data, to build the decision trees from, and the remaining data, the test data to test if the model generalizes well to new data. By building each tree from a random subset of the training data the algorithm reduces over-fitting. Using random sampling with replacement for each tree is called bootstrapping. When deciding the splits in decision trees, it is also an option to consider only a subset of the features at each split. While generally used to reduce overfitting, this may lead to poorer prediction performance when there are many (noisy) features, as it increases the chance of leaving out important features when deciding the splits [16].

It is possible to get an indication of which biological mechanisms are at play by performing feature importance analysis on a trained model. In feature importance analysis, features are ranked by their ability to predict the target. There are different ways to measure feature importance. One is to measure the impact each individual feature has on the model, by randomly permuting its feature values and measuring the effect on prediction accuracy. Another measure, impurity-decrease, is calculated directly from the trained model. It bases feature importance on how much the feature averagely decreases the variance of the training data when making a split. A third measure, SHAP-values, provide insights into the effects different feature values have on the prediction [17]. However, ranking genomic features by their importances does not automatically reveal which ones are involved in causing a phenotype. For instance, high correlation between informative features decreases their feature importances [16, 18]. This is because each tree in the RF will pick one of the correlating features at random to use in the model. Once one of the correlating features has been selected, the remaining contain no additional information and will not be used in that tree. Genomic data contains many correlated features. This dilution of feature importance means that unimportant features can seem more important than they are. Feature analysis of machine learning models therefore require careful examination to extract information about the mode of action behind the phenotype.

In this paper, the machine learning algorithm RF is explored as a high-throughput *in-silico* screening tool for predicting maximum hourly acidification rates (V_max_) of *L. lactis* strains based on their genome sequences. The strains are from two subspecies: *L. lactis* subsp. *lactis* and *L. lactis* subsp. *cremoris*. Four different genomic representations are explored for the prediction of V_max_ (genes, Pfam domains, 8-mers and 9-mers) and the resulting models are analysed with Shap [17] to evaluate whether they base their predictions on meaningful biological features. This is done by considering high-importance features with consensus across the models.

## Results

### The acidification rates of *L. lactis*

**Fig 1:**
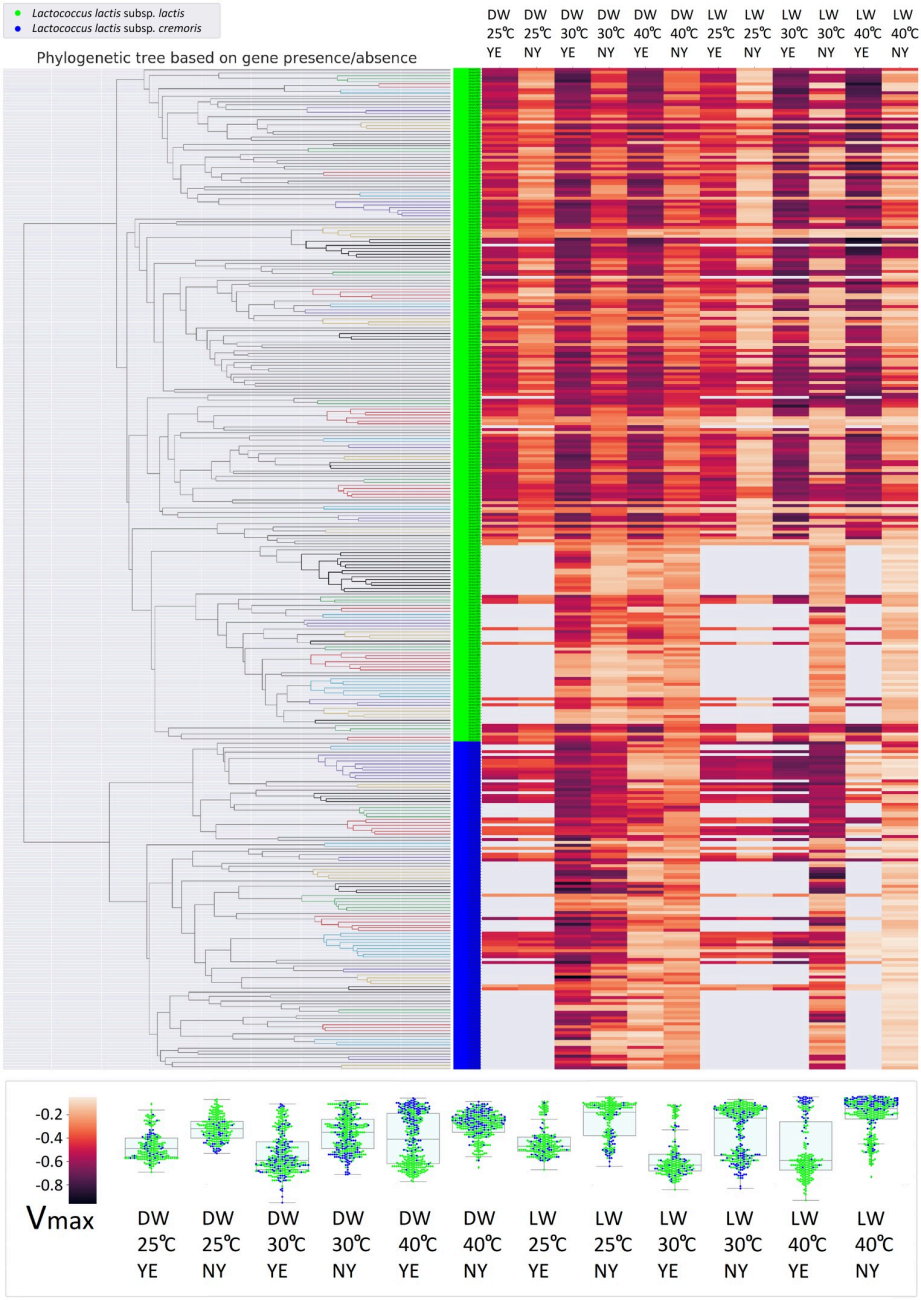
Distribution of V_max_ with regard to phylogeny. Phylogenetic tree made from clustering the gene presence/absence profiles (left) and corresponding heatmap of V_max_ (right). *L. lactis* subsp. *lactis* strains in green and *L. lactis* subsp. *cremoris* strains in blue. Each column in the heatmap shows V_max_ under a different set of conditions (DW = deepwell micro-titer plates, LW = lowwell micro-titer plates, YE = yeast extract, NY = no yeast extract). Dark purple indicate high rates of acidification and light orange indicate low rates. Missing data is indicated in grey. Box- and swarmplots (bottom) of the subspecies distribution of V_max_ for each set of conditions.

An existing dataset of maximum hourly acidification rates was obtained for 342 *L. lactis* strains grown in lowwell and deepwell micro-titer plates, with and without addition of yeast extract, and at different temperatures (25 C, 30 C, or 40 C). The pH was measured every 6 minutes during milk fermentations, and maximum hourly acidification rates (V_max_) were calculated. Since the pH of the media decreases during fermentation, acidification rates are negative. The highest observed acidification rate was -0.96 hour^-1^ and the lowest -0.05 hour^-1^. The strains tended to grow better in the presence of yeast extract (Fig 1). Both subspecies had a temperature preference for 30 C, although *L. lactis* subsp. *lactis* also had high acidification rates at 40 C in the presence of yeast extract (Fig 1).

### The *L. lactis* pangenome

**Fig 2:**
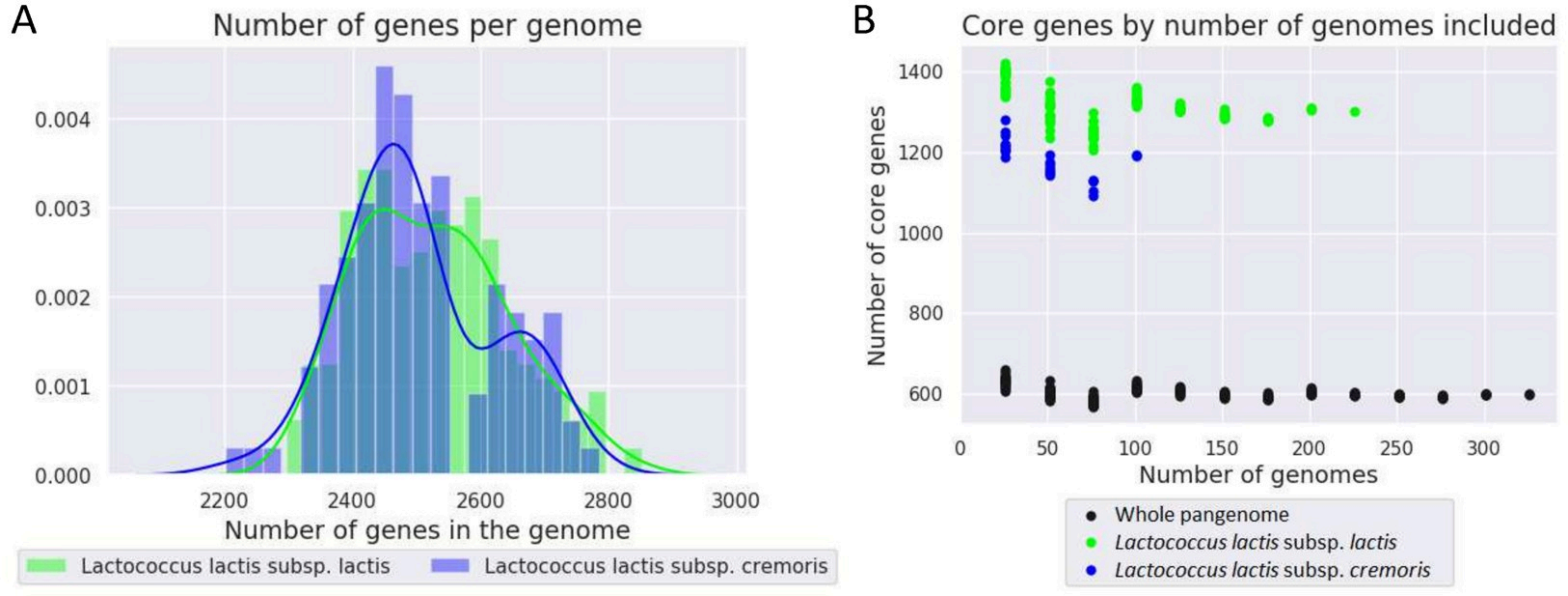
Overview of core genes and total genes in the *L. lactis* pangenome. **A.** Histogram and kernel density estimates for the number of gene groups per strain. **B.** Core genome for different size pangenomes. The core genome is defined as the set of genes, which occur in 99% of the strains or more. For each 100 strains added, a core gene is therefore allowed to be missing in 1 (more) genome. *L. lactis* subsp. *lactis* in green and for *L. lactis* subsp. *cremoris* in blue.

The 342 *L. lactis* strains were genome sequenced. There were 230 *L. lactis* subsp. *lactis* strains, and 112 *L. lactis* subsp. *cremoris* strains. The strains contained between 2200 and 2800 genes each with no discernible difference between the two subspecies (Fig 2A).

To establish the presence/absence of genes in the strains, and to get an overview of the data, a pangenome was constructed using Roary [19]. A high similarity threshold in Roary for clustering genes together (95% identity) resulted in few core genes and different gene variants being represented in separate gene groups.

Table 1 shows the distribution of genes into core genes (genes occurring in 99% of the strains or more), soft core genes (occurring in 95-99% of the strains), shell genes (occurring in 15-95% of the strains) and cloud genes (occurring in less than 15% of the strains). The core genome of all strains consisted of 595 genes (2.93% of the genes), whereas the core genomes of each of the two subspecies by themselves were bigger: 1295 core genes (8.64% of the genes) in *L. lactis* subsp. *lactis* and 1180 core genes (10.83% of the genes) in *L. lactis* subsp. *cremoris* due to a higher similarity within a subspecies than between two subspecies (Table 1). The core genomes stabilized quickly (Fig 2B). Despite having more strains in the pangenome, the *lactis* subspecies had more core genes than the *cremoris*. Only 5585 genes were found in strains of both subspecies.

**Table 1:** Pangenome gene distribution.

	*L. lactis*, both subspecies (342 strains)		*L. lactis* subsp. *lactis* (230 strains)		*L. lactis* subsp. *cremoris* (112 strains)	
	Count	Percent	Count	Percent	Count	Percent
Core genes (in ≥99% of strains)	595	2.93	1295	8.64	1180	10.83
Soft core genes (in 95%-99% of strains)	83	0.41	190	1.27	136	1.25
Shell genes (in 15%-95% of strains)	3556	17.52	1789	11.94	2124	19.50
Cloud genes (in <15% of strains)	16062	79.14	11715	78.16	7452	68.42
Total genes	20296	100.00	14989	100.00	10892	100.00

The distribution of core genes, soft core genes, shell genes, and cloud genes in the Roary pangenome computed for all the *L. lactis* strains and in the two subspecies-pangenomes.

Clustering strains by their gene presence/absence profiles was consistent with subspecies annotations (Fig 1) as the two subspecies clustered separately. Maximum hourly acidification rates however, varied a lot between strains with similar profiles in terms of gene presence/absence.

### All four RF models predicted V_max_ well

**Fig 3:**
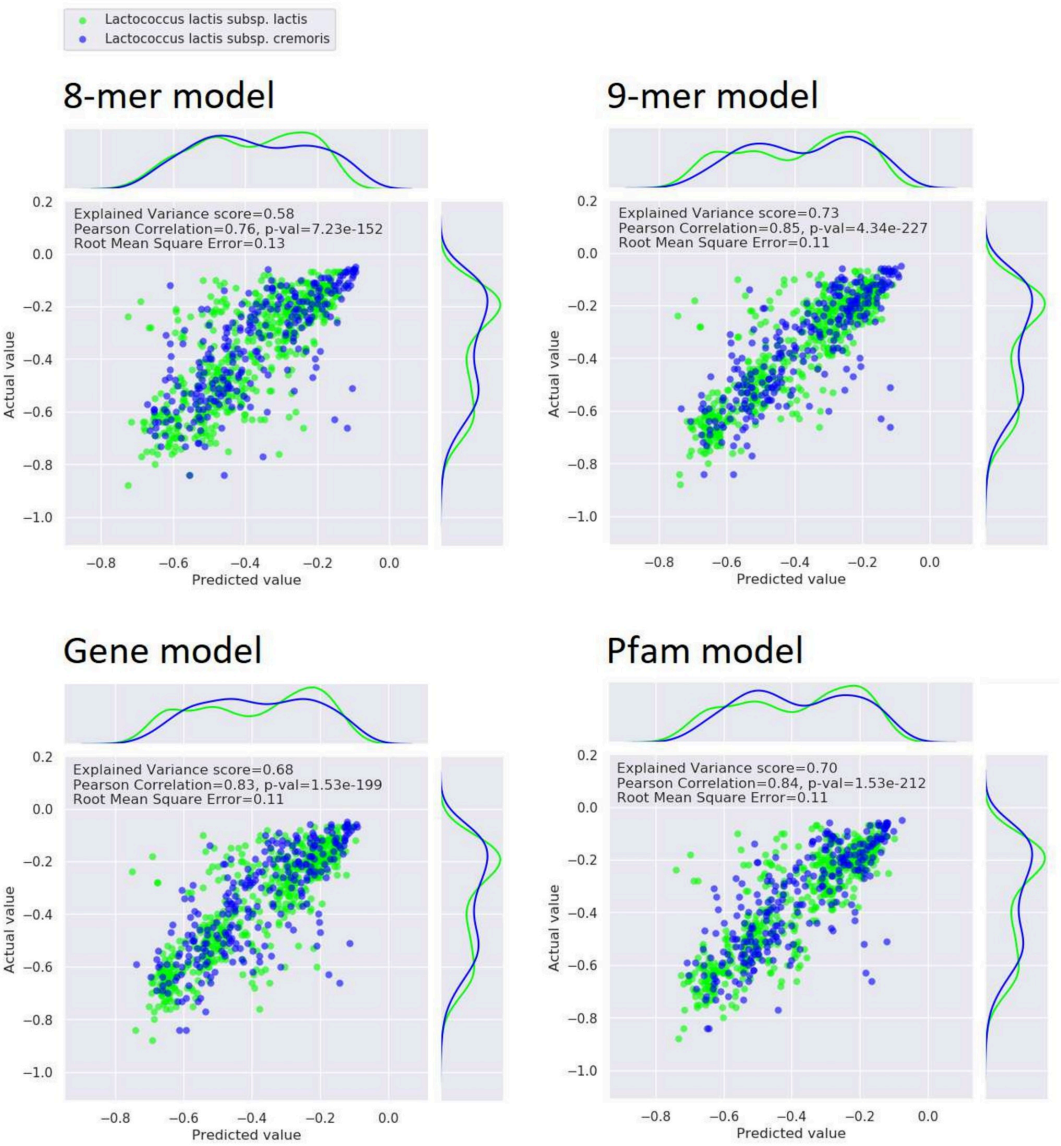
Evaluation of model predictions on test set. Predicted test set values plotted against the actual values of the maximum hourly acidification rate. Perfect predictions would produce a line where *y* = *x*. The distributions of the predicted values and the actual values are shown above and to the right of the plot respectively. *L. lactis* subsp. *lactis* strains are colored green and *L. lactis* subsp. *cremoris* strains are colored blue. For each model, three scores evaluate the accuracy of the predictions: The Explained Variance, the Pearson Correlation, and the Root Mean Square Error.

Four RF models were trained to predict V_max_ based on one of four different representations of genomic information (8-mer counts, 9-mer counts, Pfam domain counts, presence/absence of genes) and experimental variables (temperature, volume, and yeast extract addition). All models were trained on the same set of 257 strains and tested on a separate set of 85 strains. As there was up to 12 measurements for each strain, there were 2526 data points in the training set and 798 data points in the test set. For the test data, Pearson Correlation coefficients (PC) between predicted and actual V_max_ values were 0.76 (8-mer model), 0.85 (9-mer model), 0.83 (gene model), and 0.84 (Pfam model), see Fig 3. The most accurate predictions for the test data were thus achieved with the 9-mer model.

### The RF models relied on genomic features for making accurate predictions

Two tests were performed for the Pfam and gene models to find out if the models relied on genomic signals or if they based the predictions solely on the condition features (temperature, volume, and addition of yeast extract).

**Fig 4:**
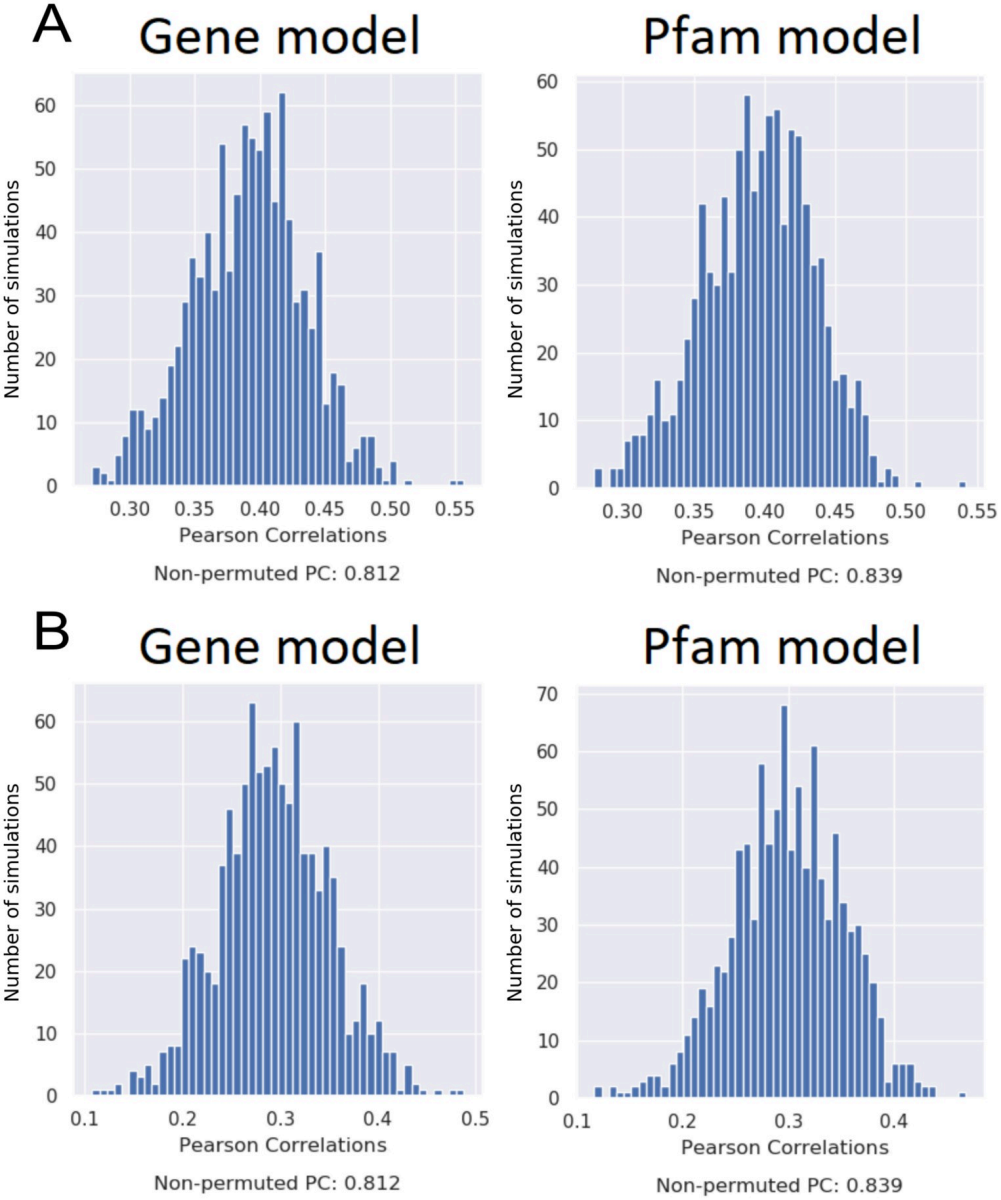
Evaluation of Pfam model and gene model reliance on genomic features. **A.** The PC scores for predictions on 1000 test sets, for which the features have each been permuted. **B.** The PC scores for predictions on 1000 test sets, for which the genomic profiles have been switched between strains.

In the first test, the values of each genomic feature of the test set were randomly permuted before obtaining a PC score for the prediction. This simulation was performed 1000 times. The PC score of the prediction for the non-permuted test data was then compared to the 1000 PC scores for permuted test sets (Fig 4A). If the model relies on genomic features to make accurate predictions, permuting the feature values randomly is expected to decrease PC scores significantly.

In the second test, genomic feature values were switched randomly between strains. This way, the relationship between features was kept intact. Again, the performance of the RF model on the non-permuted test data was compared to 1000 PC scores of permuted test sets (Fig 4B).

For both the Pfam and gene models, the PC scores in both tests were significantly higher (outside of the 95% confidence interval) for the original test data than for the permuted data, indicating that the RF models rely on a genomic signal as well as the condition features. The computation was too heavy to run for the two k-mer models, but given the results for the two other models, genetic features are expected to be important for prediction accuracy in the k-mer models as well.

### Pfam model prediction scores were stable when the model was trained on ∼75 strains or more

**Fig 5:**
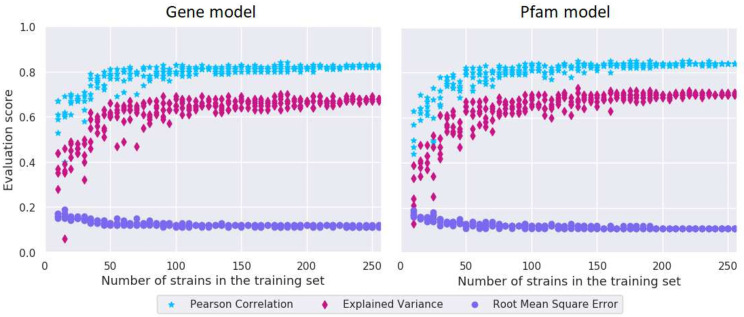
Estimation of necessary training data size. Prediction scores of the test set, when the gene model and the Pfam model was trained on different sized subsets of the training data.

The models require a certain size dataset depending on the complexity of the phenotype. Fig 5 shows how the evaluation scores depend on the number of training samples in the gene and Pfam models—i.e. how much the prediction errors for the test data change when models were built from different sized subsets of the training data. When the subset of the training data used to build the Pfam model consisted of around 75 strains, scores did not improve much by adding more strains to the training data. For the gene model, more strains were necessary to reach a stable PC score. When the model was built from around 100 strains, the scores stopped improving much with the addition of more strains. The number of strains needed for the 8-mer and 9-mer models were not computed, but are expected to be higher for the 9-mer model due to the higher number of features.

### Feature importance analysis can reveal genetic markers of higher acidification rates

Feature importance analyses were performed to find out which genes, Pfam domains and k-mers were identified as predictors of higher acidification rates. If acidification rates were strongly associated with phylogeny, the model might make good predictions based on genes which indicated the phylogeny but were not involved in causing higher acidification rates. The association of phylogeny and V_max_ was therefore first evaluated in Fig 1. Both high and low V_max_ values are represented by both subspecies, and strains next to each other in the phylogenetic tree have different V_max_ values. The phylogenetic tree is based on clustering of gene presence/absence profiles of the strains. The figure thus shows that V_max_ does not associate strongly with phylogeny.

Although phylogeny did not directly determine phenotype, feature importance analysis was still expected to turn up many noisy features. This was due to the number of features being much greater than the number of V_max_ observations, and due to the highly structured feature-space (many correlations between features because of population structure). To filter out noise from the feature importance analyses, high importance features in three models were compared. In the feature importance analyses we focus on consensus features—i.e. features with high importances in more than one model. However, features from the models do not correspond one to one. The same Pfam domain can occur in many different genes, and a 9-mer can not only be found in many different genes, but can also correspond to regions of the DNA outside of genes. Since the k-mers were calculated from genome sequences, each k-mer was counted together with its reverse-complement.

**Fig 6:**
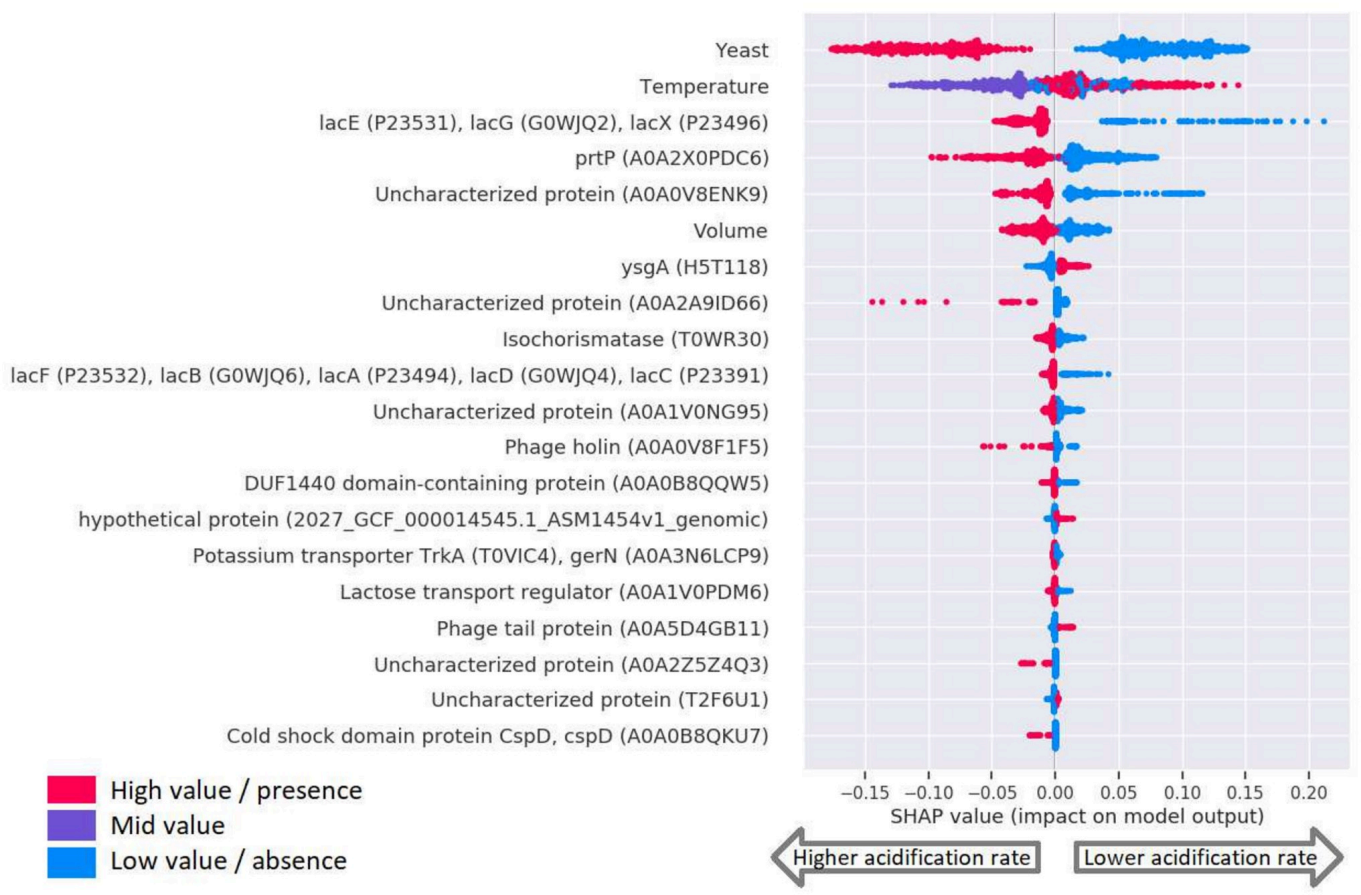
The 20 features with highest feature importances in the gene model. Highest 20 feature importances as calculated by Shap [17]. The SHAP values indicate each feature value’s impact on the prediction value. SHAP values are plotted for each feature, for each prediction. The color indicates the feature value. Depending on the feature, red means either presence of the gene(s), a temperature of 40°C, presence of yeast extract, or experiment performed in deepwell micro-titer plates. Purple means a temperature of 30°C. Blue means absence of the gene(s), a temperature of 25°C, absence of yeast extract, or experiments performed in lowwell micro-titer plates. A negative SHAP value indicates that the feature for this data point impacted the prediction towards a higher acidification rate. Features which always co-occur are grouped together previous to building the RF model. Uniprot IDs are given in parenthesis when available, otherwise the IDs from the pangenome.

The feature analysis was performed with Shap [17]. Features with the highest importances are shown in Fig 6 (gene model), Table 2 and S1 Fig (Pfam model), Table 3 and S2 Fig (9-mer model), and S3 Fig (8-mer model). In the SHAP plot in Fig 6, the 20 most important features are listed in descending order. For each feature, every prediction on the test set is indicated by an individual dot. The value of the feature is indicated by color, and the effect on the prediction of having that value is shown along the x-axis. Dots further towards left indicate that having the given value pushed the prediction towards a higher acidification rate. Dots towards the right indicate that the value pushed the prediction towards lower acidification rate.

**Table 2:** The 15 features with highest feature importances in the Pfam model.

Pfam domain	Strains	Pfam domain name	Genes the Pfam domain occurs in
PF00639.16	178	Rotamase	*prtM* (*prsA*)
PF02502.13	287	LacAB_rpiB	*lacA*, *lacB*, *rpiB*
PF02254.13	185	TrkA_N	*ktrA*
PF02386.11	185	TrkH	*ktrB*
PF13493.1	165	DUF4118	*kdpD*
PF02669.10	166	KdpC	*kdpC*
PF03814.10	166	KdpA	*kdpA*
PF02702.12	165	KdpD	*kdpD*
PF07274.7	246	DUF1440	Uncharacterized proteins
PF11361.3	247	DUF3159	Uncharacterized proteins
PF01432.15	332	Peptidase_M3	*pepF*, *pepB*
PF07083.6	128	DUF1351	Uncharacterized proteins
PF12730.2	286	ABC2_membrane_4	ABC transporter permease, Lantibiotic ABC transporter permease, *nisG*, Uncharacterized proteins
PF05649.8	204	Peptidase_M13_N	Endopeptidase *pepO*, Oligoendopeptidase O
PF02486.14	302	Rep_trans	Replication initiation protein, Transcriptional regulator, Phosphoglucomutase, Uncharacterized proteins

The fifteen Pfam domains with the highest feature importances, the number of strains they occur in, the domain names, and the genes they occur in. Only genes which accounted for more than 10 occurrences of the Pfam domains are listed. All the Pfam domains in the table impacted the predictions towards a higher acidification rate, except ABC2_membrane_4 and Rep_trans. The full overview and SHAP plot can be found in S1 File and S1 Fig.

**Table 3:** The fifteen features with the highest feature importances in the 9-mer model.

9-mer / Reverse-complement	Genes the 9-mers occurs in
AGGGCCCAG / CTGGGCCCT	6-phospho-beta-galactosidase (*lacG*)
CCGAGACCG / CGGTCTCGG	6-phospho-beta-galactosidase (*lacG*)
	Tim44 domain-containing protein
AAGATCTAC / GTAGATCTT	Glutamate synthase large subunit
	3’-5’ exoribonuclease YhaM (*yhaM*)
	Cytochrome c-type biosis protein DsbD protein-disulfide reductase
	Sensor histidine kinase KdpD (*kdpD*)
	Helix-turn-helix transcriptional regulator
GCCGTCGAC / GTCGACGGC	PIII-type proteinase (*prtP*)
AGAGTCCGG / CCGGACTCT	PTS system lactose-specific EIICB component (*lacE*)
CCGGGTAGC / GCTACCCGG	PIII-type proteinase (*prtP*)
GCCAGGGAC / GTCCCTGGC	PIII-type proteinase (*prtP*)
CTACCCGGC / GCCGGGTAG	PIII-type proteinase (*prtP*)
CGCGGCGTA / TACGCCGCG	PIII-type proteinase (*prtP*)
CGCCGCGGC / GCCGCGGCG	PIII-type proteinase (*prtP*)
	Copper/potassium-transporting ATPase (*copA*)
CGCAGCCCC / GGGGCTGCG	CBS domain-containing protein (*ytoI*)
	HAD family hydrolase
	Teichoic acid biosynthesis protein (*tagZ*)
	Citrate lyase alpha chain (*citF*)
	3-isopropylmalate dehydrogenase (*leuB*)
	N-acetyl-gamma-glutamyl-phosphate reductase (*argC*)
ACTGGGCCC / GGGCCCAGT	6-phospho-beta-galactosidase (*lacG*)
	Glyoxalase
	Glyoxalase family protein
GATCTAACC / GGTTAGATC	Cation transporter
	Site-specific recombinase (*orf30*)
	Orotidine 5’-phosphate decarboxylase (*pyrF_1*)
	DNA-invertase hin (*hin_1*)
CGGAACCCG / CGGGTTCCG	Diphosphomevalonate decarboxylase
CGACCTACA / TGTAGGTCG	Potassium uptake protein, integral membrane component (*ktrB*)
	Molecular chaperone GroES
	Probable potassium transport system protein kup 2 (*kup2*)

The fifteen 9-mers with the highest feature importances and the genes they occur in. All k-mers are counted together with their reverse-complement previous to building the RF model. Gene annotations are only given for genes which the 9-mer occurs in for more than 40 strains. All 9-mers in the table impacted the predictions towards a higher acidification rate, except CGCAGCCCC/GGGGCTGCG and CGGAACCCG/CGGGTTCCG. The full overview and SHAP plot can be found in S2 File and S2 Fig.

### Yeast, temperature, and volume were high importance features in all four models

All four models identified addition of yeast extract to the media as the most important feature. Since many *L. lactis* strains grew better in the presence of yeast extract, it makes sense that this would be an important feature. Temperature is likewise known to impact acidification rates [20]. Growth in deepwell micro-titer plates also resulted in higher acidification rates than growth in lowwell micro-titer plates, likely because of higher oxygen levels in lowwell plates than in deepwell plates [21].

### Genes for lactose metabolism were identified as predictors of higher acidification rates

The gene model indicated the *lacABCDFEGX* genes as indicators of higher acidification rate (Fig 6). The *lac* genes of *L. lactis* are responsible for the tagatose 6-phosphate pathway (*lacABCD*), the phosphotransferase system (*lacF* and *lacE*), the phospho-beta-galactosidase (*lacG*), and one unknown function (*lacX*) [22, 23]. These genes were split into two features in the gene model, since the *lacABCDF* genes were missing in strain354 and the *lac* genes therefore did not co-occur in all strains. The *lacEGX* genes were at the end of a contig in strain354 and the strain acidified milk, so the *lacABCDF* genes were likely missing due to an assembly error. However, both the *lacABCDF* and the *lacEGX* genes were indicated by the feature importance analysis (Fig 6). The Pfam model identified the LacAB_rpiB domain (PF02502) as an indicator of higher acidification rate (Table 2). This domain was present in both the *lacA* and *lacB* genes as well as in another gene, *rpiB*. Among the fifteen 9-mers with highest feature importances, three 9-mers found in *lacG* and one 9-mer found in *lacE* were indicated as markers of higher acidification rate (Table 3).

### Genes for casein degradation were identified as predictors of higher acidification rates

The gene model identified the serine proteinase PrtP as an indicator of higher acidification rate. The 9-mer model also indicated *prtP* through six of the fifteen most important 9-mers (Table 3). In its matured form, PrtP degrades casein [24]. Caseins make up ca. 80% of the protein in bovine milk [25]. The free amino acid and small peptide content in milk can only account for up to 20% of the final biomass of *Lactococcus* grown in milk, the remaining peptides and amino acids required come from the hydrolysis of casein [1]. Matured PrtP degrades caseins into large oligopeptides of 4-30 residues [1]. The oligopeptides of 4-18 residues are then transported by the oligotransport system, Opp, into the cytoplasm to be degraded by peptidases [25]. The Opp system is present in all the *L. lactis* strains of this study (Uniprot ID T2F365), so the models did not identify the Opp genes.

After transport into the cell, the oligopeptides are degraded by peptidases [1, 25]. The Pfam model identified Peptidase_M3 (PF01432) as an indicator of higher acidification rate. This domain was found in the oligoendopeptidase *pepF* and the oligopeptidase *pepB*.

The Pfam model identified *prsA* as an indicator of higher acidification rate through the Pfam domain PF00639 (Rotamase). This gene is also called *prtM*, and it encodes the maturation protein for PrtP. The *prtM* gene was not indicated by the gene model as it had been split into multiple gene groups in the pangenome. PrtP on the other hand was not identified through the Pfam domains with highest feature importance. The protein contains 4 Pfam domains: Peptidase_S8 (PF00082), PA (PF02225), DUF1034 (PF06280), and Gram_pos_anchor (PF00746). While two of these were found in many other genes (S4 File), the other two were primarily found in PrtP. However, as the Rotamase domain only occurred in PrtM, this may have resulted in Rotamase being the better predictor.

### Genes involved in potassium transport were identified as predictors of higher acidification rates

The potassium-transporting ATPase system Kdp and the potassium uptake system Ktr were both indicated as predictors of higher acidification rate. In the Pfam model, the two domains with the highest feature importances were domains found in *lacAB* and *prtM*. After these, the next six Pfam domains were all from genes in the Kdp and Ktr systems (Table 2). The Ktr system was also indicated by the *ktrA* gene in the feature importance analysis of the gene model, and *kdpD* and *ktrB* were indicated through the 9-mers AAGATCTAC/GTAGATCTT and CGACCTACA/TGTAGGTCG respectively.

These systems could be involved in keeping intracellular pH-levels from becoming too low during the production of lactic acid. *L. lactis* strains can grow in relatively acidic environments by keeping the intracellular environment at a near neutral pH [26]. Decrease in the intracellular pH has been shown to make the cells increase the exchange of K^+^ for protons [27]. Potassium-proton exchange is involved in pH homeostasis in other bacteria [28, 29]. We therefore suggest that the *kdpACD* and *ktrAB* genes could be involved in maintaining a neutral intracellular pH by functioning as ATP-driven antiporters, which pump the positively charged potassium ions into the cell and the negatively charged hydrogen out of the cell against the concentration gradient.

The Kdp system was only present in the *L. lactis* subsp. *lactis* strains, not in the *L. lactis* subsp. *cremoris* strains. However, it was not present in all *L. lactis* subsp. *lactis* strains. Out of 230 *L. lactis* subsp. *lactis* strains, 165 carried both the *kdpA*, *kdpC*, and *kdpD* genes, and one strain contained only the *kdpC* and *KdpD* genes. This indicates that the model does not use the genes as a proxy for subspecies.

Other systems for pH homeostasis in *L. lactis* include the F_1_F_0_-ATPase complex [30], arginine deiminase pathway [31, 32], malolactic fermentation [31, 32], and glutamate decarboxylation [31, 32]. Each system was found in all or nearly all of the strains (S11 File), and they were not indicated as highly predictive of higher acidification rates by feature importance analysis of either of the models.

### Uncharacterized genes and domains of unknown function were identified as important features

An uncharacterized gene was indicated by both the gene model and the Pfam model. This was the DUF1440 domain-containing protein with Uniprot ID A0A0B8QQW5. There were also many genes, both characterized and uncharacterized, which were only indicated by one of the models. Although some of these may be important features that were only detectable by the RF through that particular genomic representation, many of them are possibly noise.

## Discussion

Four genomic representations were explored for the prediction of maximum hourly acidification rates of *L. lactis* strains with Random Forest (genes, Pfam domains, 8-mers, and 9-mers). All four RF models predicted the maximum hourly acidification rates with high accuracy and did so by identifying relevant genomic signals. The PC scores for the correlation between observed and predicted V_max_ were 0.83 (gene model), 0.84 (Pfam model), 0.76 (8-mer model), and 0.85 (9-mer model) in a test set. Of the four models, the 9-mer model thus had the highest correlation between actual and predicted acidification rates. This may be because the 9-mer model does not rely on annotation or on a clustering step to define the features. In the gene clustering procedure of the pangenome, some genes with the same function were not grouped together. This was partly due to the high identity threshold, partly due to the nature of the heuristic method. For example, the *prtM* genes were split into multiple gene groups and the gene was thus not recognised as the same entity in the Random Forest. In this case, the model identified the *prtP* gene instead, but for other mechanisms there might not be an alternative signal for the model to identify. This was for instance the case for the Kdp system, for which all the genes were split into multiple gene groups.

The Pfam model however performed similarly to the 9-mer model. In theory, using Pfam domains would likely give a better performance when building models of more distantly related strains, since the nucleotide sequences of genes would be expected to have remained less conserved between such strains.

The 9-mer model performed much better than the 8-mer model—likely because 8-mers were too short to map uniquely to relevant regions of the genomes. The k-mer models have the potential to identify functionally important regions of the DNA other than those which encode genes. They also have the potential to distinguish between genes with minor mutations, whereas the gene and Pfam models would only be able to distinguish between genes with larger mutations. However, k-mer models can have a very large number of features for higher values of k, most of which are noisy (non-informative). Identifying the functionally important regions requires that at least one k-mer in such a region is both unique to the region and well conserved. While using longer k-mers increases the chances for finding k-mers unique to a region, it also increases the number of features drastically, making the models computationally expensive.

Prediction scores stabilized when including 75 strains or more in the training set. Subspecies-specific models were therefore not investigated as there was not enough data to both build and test them. However, feature importance analysis of the models containing data from both subspecies identified both subspecies-specific and shared mechanisms for higher acidification rates.

This study shows, that without any previous knowledge of which genes influence the phenotype, it is possible with machine learning to obtain an accurate picture of the mechanisms at play and to learn which genes are more indicative of a phenotype. The high importance features were in line with previous knowledge of important processes in milk acidification. Genes for both casein degradation and lactose metabolism were identified, which is supported by literature. Genes for potassium transport were also identified. These genes are possibly part of a stress response to keep intracellular pH within a normal range as the external pH drops during acidification. *L. lactis* has been shown to increase the exchange rate of extracellular potassium ions for intracellular protons to increase intracellular pH during low extracellular pH [27]. One of the potassium transport systems, Kdp, was only found in *L. lactis* subsp. *lactis*. This may be part of the reason for the generally higher acidification rates of the subspecies compared to that of *L. lactis* subsp. *cremoris*. Some genes indicated by the feature importance analyses were however of unknown function. These should be investigated further to learn their exact role in the acidification process.

This study shows that it is possible to use machine learning to predict a complex bacterial phenotype from whole genome sequencing data. With careful attention to the model mechanisms and correlated features, it is also possible to identify the key mechanisms driving that phenotype. This can prove valuable in many other areas besides cheese-making. It can give highly useful indications of which genes to examine for poorly-understood phenotypes such as texture-formation in yoghurt by lactic acid bacteria, immune response to probiotics, or pathogen-inhibition by non-pathogenic bacteria. It can be used for large-scale screening of new bacteria, and prove especially useful for predicting phenotypes of strains that are difficult to grow—such as many anaerobic bacteria.

## Materials and methods

### Acidification assay

Milk acidifications were conducted as described in [2], with minor modifications. In short, strains were retrieved from -80 C glycerol stocks, thawed, and 20 μl were transferred to 180 μl M17 broth [33] enriched with 1% glucose and 1% lactose and incubated overnight at 30 C. The outgrown cultures were diluted 100 fold in milk with pH indicator ± yeast extract (0.2%) and 200 μl or 2000 μl were transferred to 96 well lowwell or deepwell plates, respectively. The plates were incubated on top of flatbed scanners, at 25 C, 30 C and 40 C. Measurements of pH, based on color of wells was done every 6 minutes. Converting measured hue values into pH values was done in the following manner: The color (hue) of milk with pH indicator at different pH values, ranging from pH 6.5 to pH 4.0, was measured using a HP Scanjet G4010 flat-bed scanner and a pH electrode was used to measure the pH of the same wells, to generate a calibration curve: Hue vs pH. A 4th grade polynomial fit was used to convert hue values into pH values. This fit was then used to convert all measured hue values from milk acidification experiments into pH values. V_max_ was calculated as the maximum negative slope over 1 hour (10 measurements). Vmax was typically found in the middle of the pH drop.

### DNA extraction

Extraction of DNA used for genome sequencing of short reads on the Illumina MiSeq instrument was performed using the DNeasy Blood and Tissue kit (Qiagen, Hilden, Germany). The protocol suggested by the vendor was applied with the exception that cells were initially treated with 20 mg/ml lysozyme for 30 min at 37 C followed by proteinase K treatment for 30 min at 56 C. The remaining protocol was conducted on the QIAcube instrument (Qiagen, Hilden, Germany).

### Library preparation and sequencing

For the Illumina platform DNA was fragmented using the BioRuptor instrument (Diagenode Inc., Denville, NJ, USA) aiming at an insert size of 800bp. Fragmented DNA was used as input to the KAPA HTP kit (Roche, Basel, Switzerland) and libraries were build according to the manufacturers recommendations. Sequencing was done using V2 chemistry on the MiSeq instrument (Illumina, San Diego, CA, USA).

### Assembly

Sequencing reads from the MiSeq were trimmed using the CLC genomics software with setting for quality 0.005 and for length minimum 50 bases and maximum 300 bases. Singletons were also removed. Trimmed reads were *de novo* assembled using CLC genomics software with default settings. Subsequent to the assembly, contigs with lower coverage that 50% of the average genome coverage were removed as contaminations.

### Species identification and quality control

Species and subspecies identification was done with KmerFinder [34, 35] using the bacteria.ATG database version 20190108_stable downloaded on January 31 2019. Quality control of the assemblies was done with QUAST v5.0.2 by setting an N50 threshold of 20000 nucleotides.

### Thirty-five complete RefSeq genomes

Thirty-five complete RefSeq genome sequences of *L. lactis* subspecies *lactis* and *L. lactis* subsp. *cremoris* were downloaded from NCBI (S5 File). Of these, 21 were *L. lactis* subsp. *lactis* and 14 were *L. lactis* subsp. *cremoris*. The strains were obtained from searching https://www.ncbi.nlm.nih.gov/assembly for (“Lactococcus lactis subsp. lactis”[Organism] OR “Lactococcus lactis subsp. cremoris” [Organism]) AND “Complete Genome”[Assembly level] AND “latest”[filter]. These genomes were included in the pangenome for annotation purposes, but not included in any of the analyses.

### Pangenome

The genes were predicted and annotated with Prokka version 1.14.1 [36].

Roary 3.12.0 [19] was used with default settings (minimum 95% identity for blastp) to build the pangenome for the strains based on their gff 3 files from the Prokka output. This pangenome was also used to calculate the core genomes for each of the two subspecies *lactis* and *cremoris*.

To get species-relevant annotations for the pangenome, all *Lactococcus* proteins were downloaded from Uniprot (organism:“Lactococcus”), and a BLAST protein database was created. A BLASTp search was run for representative sequences randomly selected from each gene group. The search was done with an E-value of 0.01 and all matches with identity or coverage below 50% were discarded. The pangenome included some tRNA genes. As these do not encode proteins, they were annotated by BLASTn search against the bacterial tRNA sequences of [37]. For those gene groups which did not get an annotation through this process, a sequence was selected from the 35 complete RefSeq genomes. Else, an ID was obtained from NCBI through a BLASTp search with the aforementioned criteria. For 92 gene groups containing more than six genes, no annotation could be found in either of the databases. Gene groups containing fewer genes were removed (see section on Random Forest: Data pre-processing). Gene IDs in the genomic matrix thus refer to sequences in public databases (except a numerical prefix which ensures a unique feature name).

### Pfam annotation

To annotate the strains with Pfam domains, the EMBL-EBI perl script, pfam_scan.pl (ftp://ftp.ebi.ac.uk/pub/databases/Pfam/Tools/PfamScan.tar.gz), was used with the Hidden Markov Model library, Pfam-A, from version 32.0 of the Pfam database [11].

### Calculating k-mers

Since the genome sequences in the fasta files could be from either strand, a k-mer and its reverse-complement sequence are synonymous. They were therefore counted in pairs. In the matrix, they are represented by the sequence, which comes alphabetically first.

### Random Forest: Data pre-processing

Four different genomic representations were used for the machine learning algorithm: A matrix of the presence/absence of genes (based on the Roary pangenome output), a matrix of copy numbers of the Pfam domains, and two matrices of the number of nucleotide k-mers of length 8 and 9. The rows of the matrices represented strains, and each column contained information about a specific gene, Pfam domain, or k-mer. Those genes, Pfam domains, or k-mers, which were either present in nearly all genomes (more than 98%) or absent in nearly all genomes (more than 98%), were removed from the matrices to reduce noise (pruning). This removes annotation errors as well as features with little information in a machine learning context. Genes/Pfam domains/k-mers with identical profiles for all the strains (always co-occurring) were collapsed into single columns. The conditions the strains were grown under were also used as features. This added three features to the models: Temperature, Volume, and Yeast.

### Random Forest: Training

To implement the Random Forest, the RandomForestRegressor module of the python module Scikit-learn [38] was used. The data set was randomly split into a training set of 257 strains (75% of the strains) and a test set of 85 strains (25% of the strains). A random state has been set in the code to ensure deterministic behaviour.

Randomized 3-fold cross-validated grid-search for optimal parameters in the Random Forest (RF) was performed on the training data. The grid was

- n_estimators=[10,50,100,150,200,250,…,750]
- max_depth=[None,10,20,30,40,…,150]
- min_samples_split=[2,4,6,..,16]
- min_samples_leaf=[1,2,3,…,9]
- oob_score = [True, False]

The max_features parameter was kept to the default auto since there was a very large number of features, many of which were noisy. The bootstrap parameter was also left at the default True to use sampling with replacement. The parameters of the gene model found by randomized 3-fold cross validated grid-search were ‘n_estimators’: 450, ‘max_depth’: None, ‘min_samples_split’: 2, ‘min_samples_leaf’: 1, ‘oob_score’: True for the gene model. For the Pfam model they were ‘n_estimators’: 650, ‘max_depth’: 70, ‘min_samples_split’: 6, ‘min_samples_leaf’: 4, ‘oob_score’: False. For the 8-mer model they were ‘n_estimators’: 650, ‘max_depth’: 110, ‘min_samples_split’: 4, ‘min_samples_leaf’: 2, ‘oob_score’: False. For the 9-mer model they were ‘n_estimators’: 550, ‘max_depth’: 120, ‘min_samples_split’: 4, ‘min_samples_leaf’: 1, ‘oob_score’: False. The models were trained on the training data using the identified optimal parameters.

### Pipeline

Scripts and software requirements can be found at https://github.com/signetang/bacterial_phenotype_genotype_matching_RFR.

## Supporting information

S1 Fig The 20 features with highest feature importances in the Pfam model. Made using Shap [17]. The SHAP values indicate each feature value’s impact on the prediction value. SHAP values are plotted for each feature, for each prediction. The color indicates the feature value. Red means a higher value and blue means a lower value. A positive SHAP value indicates that the feature for this data point impacted the prediction towards a higher value (corresponding to a lower acidification rate). Features which always co-occur are grouped together previous to building the RF model.
(TIF)

S2 Fig The 20 features with highest feature importances in the 9-mer model. Made using Shap [17]. The SHAP values indicate each feature value’s impact on the prediction value. SHAP values are plotted for each feature, for each prediction. The color indicates the feature value. Red means a higher value and blue means a lower value. A positive SHAP value indicates that the feature for this data point impacted the prediction towards a higher value (corresponding to a lower acidification rate). Features which always co-occur are grouped together previous to building the RF model.
(TIF)

S3 Fig The 20 features with highest feature importances in the 8-mer model. Made using Shap [17]. The SHAP values indicate each feature value’s impact on the prediction value. SHAP values are plotted for each feature, for each prediction. The color indicates the feature value. Red means a higher value and blue means a lower value. A positive SHAP value indicates that the feature for this data point impacted the prediction towards a higher value (corresponding to a lower acidification rate). Features which always co-occur are grouped together previous to building the RF model.
(TIF)

S4 Fig Evaluation of model on training set. Predicted training set values plotted against the actual values of the maximum hourly acidification rate. Perfect predictions would produce a line where *y* = *x*. The distributions of the predicted values and the actual values are shown above and to the right of the plot respectively. *L. lactis* subsp. *lactis* strains are colored green and *L. lactis* subsp. *cremoris* strains are colored blue. For each model, three scores evaluate the accuracy of the predictions: The Explained Variance, the Pearson Correlation, and the Root Mean Square Error.
(TIF)

S1 File The Pfam domains with the highest feature importances and the genes in which they occur. (PDF)

S2 File The 9-mers with the highest feature importances and the genes in which they occur. (PDF)

S3 File The 8-mers with the highest feature importances and the genes in which they occur. (PDF)

S4 File The four Pfam domains in PrtP and the genes in which they were present. (PDF)

S5 File Names of complete RefSeq genomes. (PDF)

S6 File Gene matrix. Input matrix for the Random Forest. This text file contains 6759 semicolon-separated columns in 343 lines. The first line is a header. The first column contains the strain IDs, the second column contains the subspecies annotations, and the following columns contain presence/absence of genes (1s and 0s). A column can represent multiple genes if their occurrence profiles are identical—in those cases the IDs in the column header are comma-separated.
(TXT)

S7 File Pfam matrix. Input matrix for the Random Forest. This text file contains 563 semicolon-separated columns in 343 lines. The first line is a header. The first column contains the strain IDs, the second column contains the subspecies annotations, and the following columns contain counts of Pfam domains. A column can represent multiple Pfam domains if their occurrence profiles are identical—in those cases the IDs in the column header are comma-separated.
(TXT)

S8 File 8mer matrix. Input matrix for the Random Forest. This text file contains 774 semicolon-separated columns in 343 lines. The first line is a header. The first column contains the strain IDs, the second column contains the subspecies annotations, and the following columns contain counts of reverse-complement 8-mer pairs. A column can represent multiple such pairs if their occurrence profiles are identical—in those cases the IDs in the column header are comma-separated. Only the alphabetically first 8-mer in the pairs are used in the column headers.
(TXT)

S9 File 9mer matrix. Input matrix for the Random Forest. This text file contains 12615 semicolon-separated columns in 343 lines. The first line is a header. The first column contains the strain IDs, the second column contains the subspecies annotations, and the following columns contain counts of reverse-complement 9-mer pairs. A column can represent multiple such pairs if their occurrence profiles are identical—in those cases the IDs in the column header are comma-separated. Only the alphabetically first 9-mer in the pairs are used in the column headers.
(TXT)

S10 File Phenotype matrix. Input matrix for the Random Forest. This file contains 13 semicolon-separated columns in 243 lines. The first line is a header. The first column contains the strain IDs and the following columns contain V_max_ values under the different conditions.
(CSV)

S11 File Genes for pH regulation in *L. lactis*, which did not show up among the most important features in feature importance analysis. Gene names and corresponding Uniprot IDs found in the strains.
(PDF)
