# Supplementary figures and images for: Machine learning predicts and provides insights into milk acidification rates of Lactococcus lactis
Source: PLoS One. 2021 Mar 15;16(3):e0246287. doi: 10.1371/journal.pone.0246287 (PMC7959382; doi:10.1371/journal.pone.0246287)

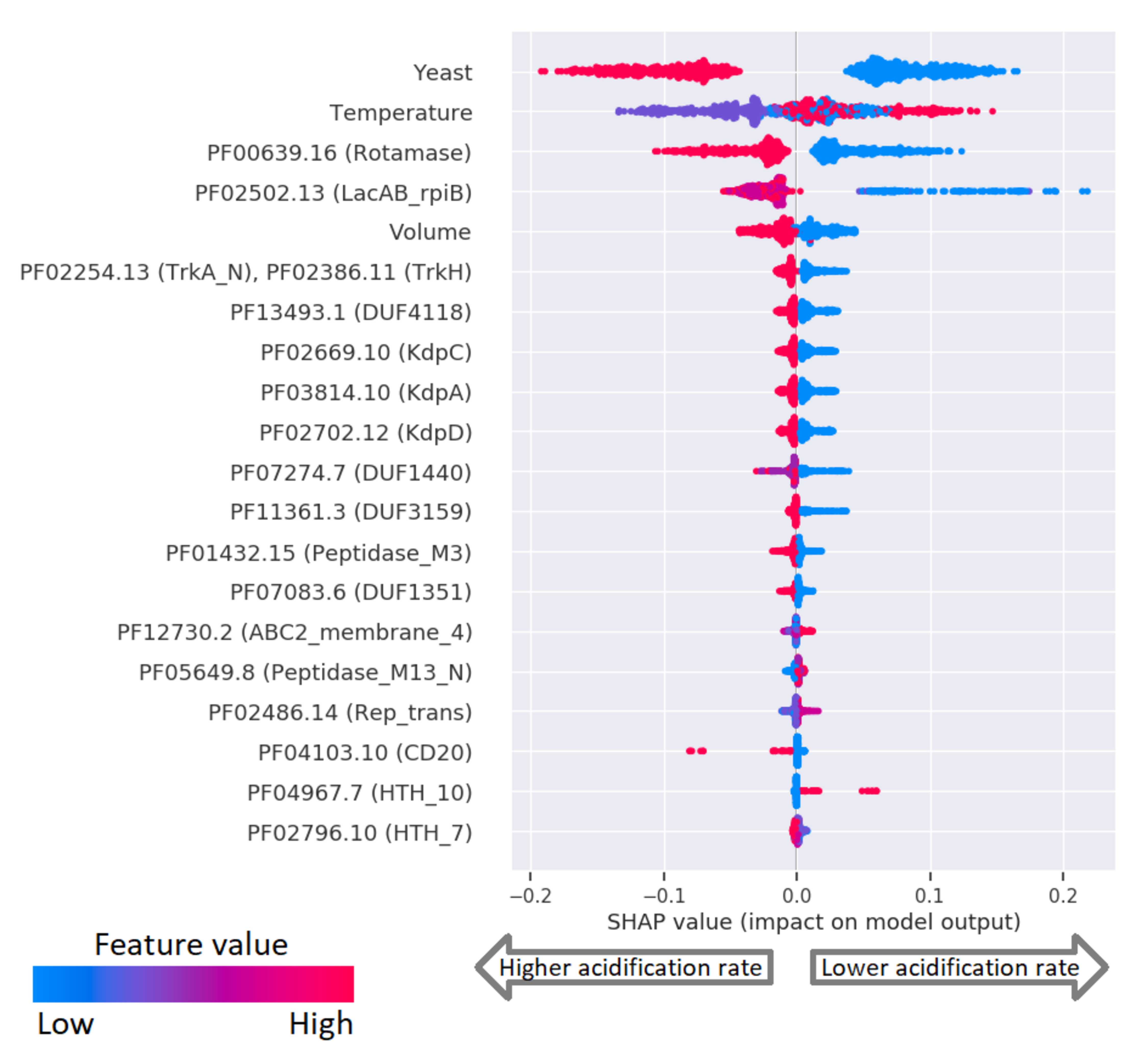

Supplement: S1 Fig — Made using Shap [17]. The SHAP values indicate each feature value’s impact on the prediction value. SHAP values are plotted for each feature, for each prediction. The color indicates the feature value. Red means a higher value and blue means a lower value. A positive SHAP value indicates that the feature for this data point impacted the prediction towards a higher value (corresponding to a lower acidification rate). Features which always co-occur are grouped together previous to building the RF model. (TIF) [file pone.0246287.s001.tif]

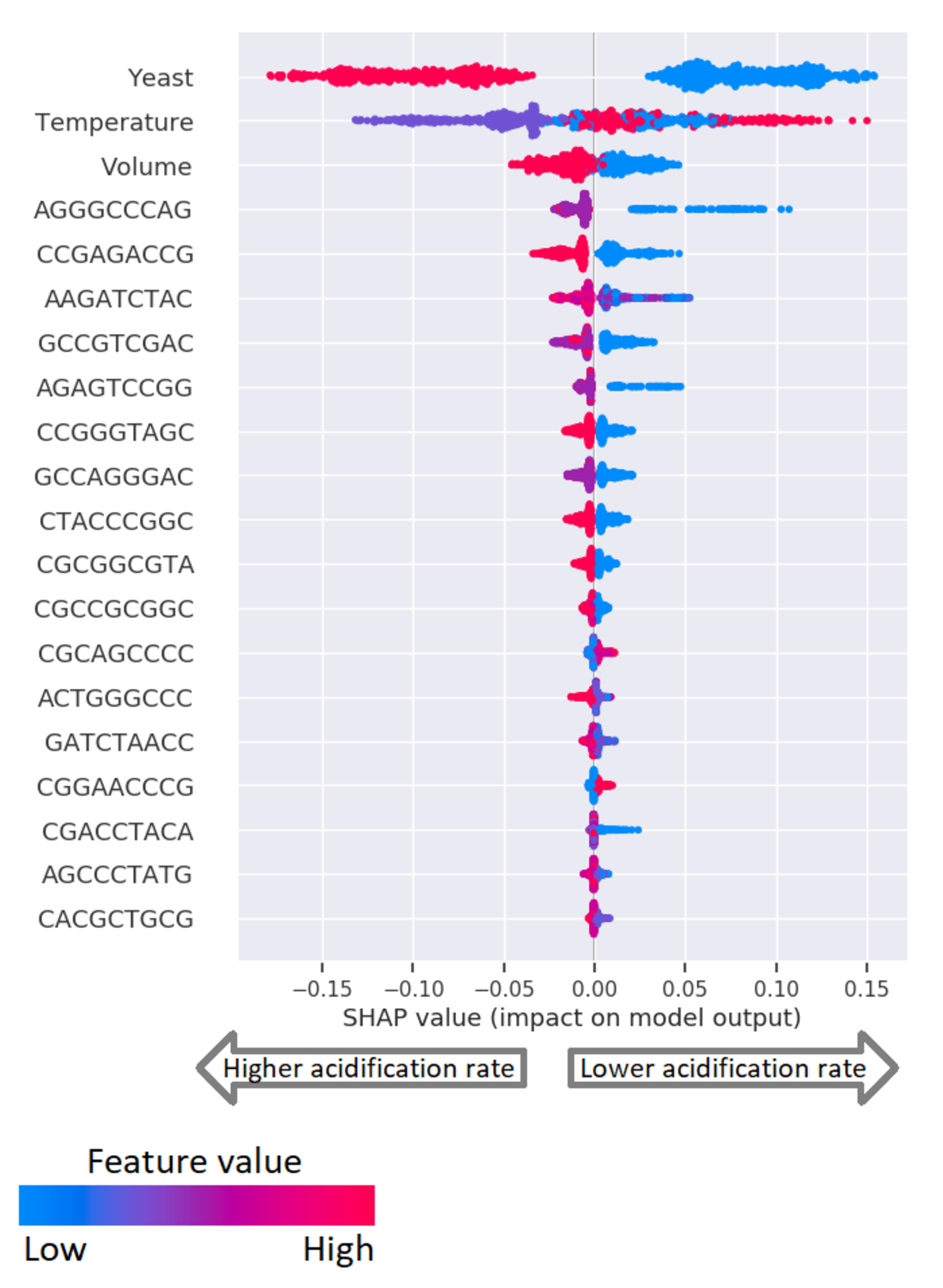

Supplement: S2 Fig — Made using Shap [17]. The SHAP values indicate each feature value’s impact on the prediction value. SHAP values are plotted for each feature, for each prediction. The color indicates the feature value. Red means a higher value and blue means a lower value. A positive SHAP value indicates that the feature for this data point impacted the prediction towards a higher value (corresponding to a lower acidification rate). Features which always co-occur are grouped together previous to building the RF model. (TIF) [file pone.0246287.s002.tif]

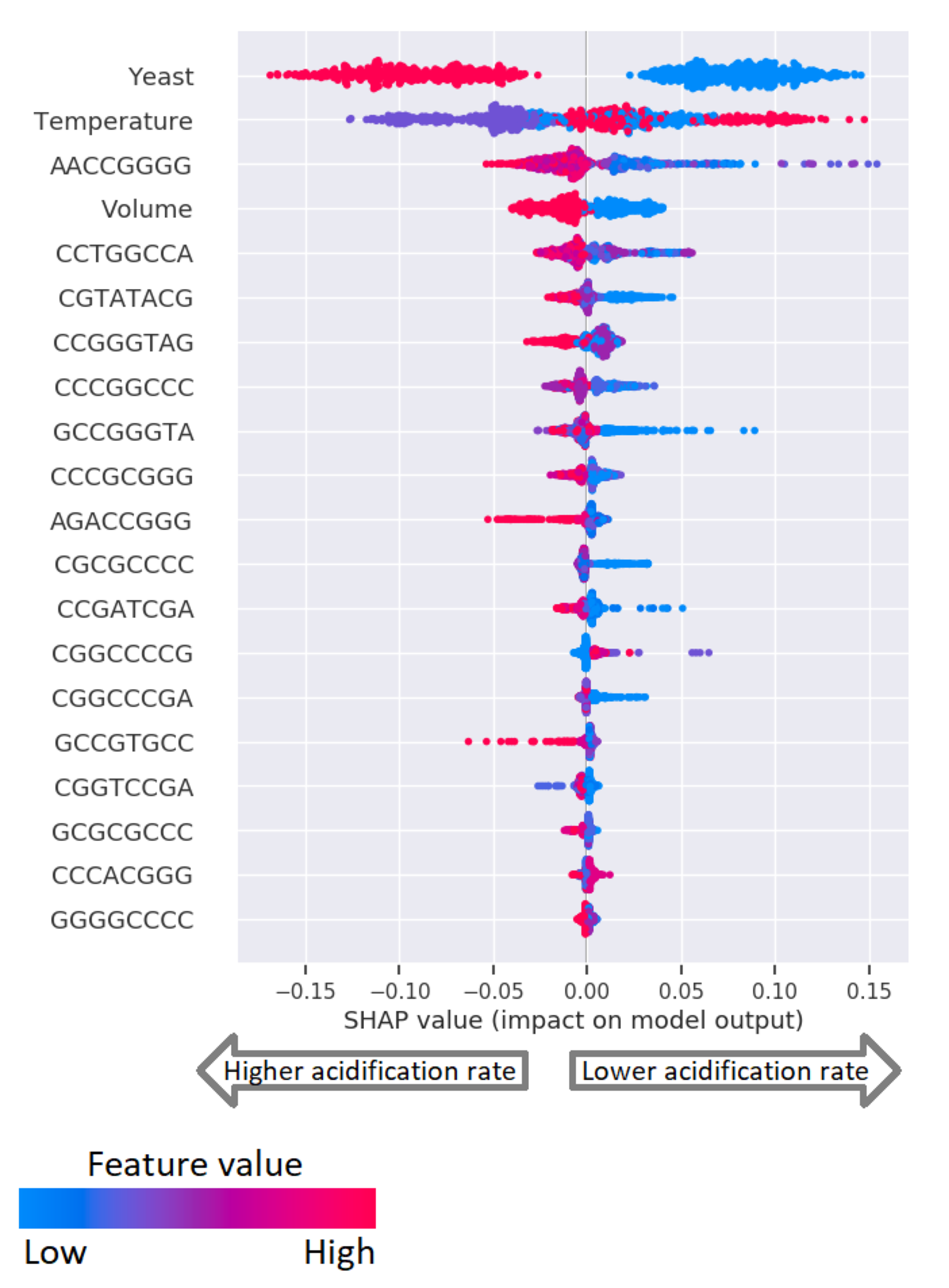

Supplement: S3 Fig — Made using Shap [17]. The SHAP values indicate each feature value’s impact on the prediction value. SHAP values are plotted for each feature, for each prediction. The color indicates the feature value. Red means a higher value and blue means a lower value. A positive SHAP value indicates that the feature for this data point impacted the prediction towards a higher value (corresponding to a lower acidification rate). Features which always co-occur are grouped together previous to building the RF model. (TIF) [file pone.0246287.s003.tif]

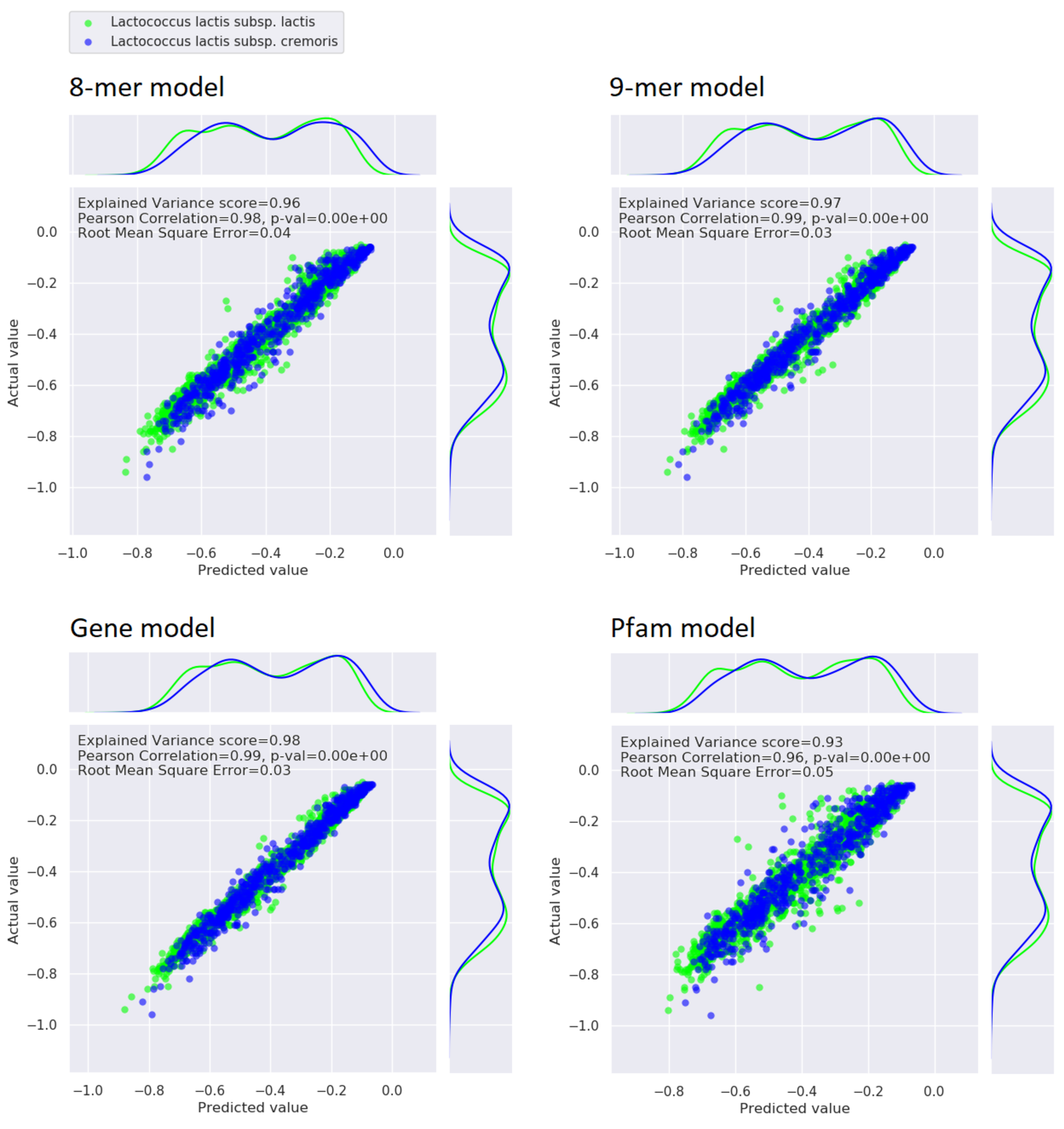

Supplement: S4 Fig — Predicted training set values plotted against the actual values of the maximum hourly acidification rate. Perfect predictions would produce a line where y = x. The distributions of the predicted values and the actual values are shown above and to the right of the plot respectively. L. lactis subsp. lactis strains are colored green and L. lactis subsp. cremoris strains are colored blue. For each model, three scores evaluate the accuracy of the predictions: The Explained Variance, the Pearson Correlation, and the Root Mean Square Error. (TIF) [file pone.0246287.s004.tif]
