# Supplementary material for: Machine learning predicts and provides insights into milk acidification rates of Lactococcus lactis
Source: PLoS One. 2021 Mar 15;16(3):e0246287. doi: 10.1371/journal.pone.0246287 (PMC7959382; doi:10.1371/journal.pone.0246287)
Supplement: S1 File — (PDF) [file pone.0246287.s005.pdf]

The Pfam domains with the highest feature importances and the genes in which they occur

```
PF00639.16
3153_T0UR35, prsA, Foldase protein PrsA         100
4201_Q02VE3, prsA, Foldase protein PrsA          44
6605_A0A0V8ESF4, prsA, Foldase protein PrsA      21
8347_Q02VE3, prsA, Foldase protein PrsA          12
13975_A0A0V8ESF4, prsA, Foldase protein PrsA      3

PF02502.13
759_G0WJQ6, lacB, Galactose-6-phosphate isomerase subunit LacB    280
760_P23494, lacA, Galactose-6-phosphate isomerase subunit LacA    280
2768_A2RJU5, rpiB, Ribose 5-phosphate isomerase B                 109
21995_A0A084ACR2, U725_00770, Ribose-5-phosphate isomerase          1
21993_A0A084ACR2, U725_00770, Ribose-5-phosphate isomerase          1
21994_A0A084ACR2, U725_00770, Ribose-5-phosphate isomerase          1
21992_A0A084ACR2, U725_00770, Ribose-5-phosphate isomerase          1
21991_A0A084ACR2, U725_00770, Ribose-5-phosphate isomerase          1

PF02254.13
1793_U6EQS3, BN927_01504, Potassium uptake protein, integral membrane component, KtrA    185
dtype: int64

PF02386.11
1798_U6ERA5, BN927_01503, Potassium uptake protein, integral membrane component, KtrB     184
14146_U6ERA5, BN927_01503, Potassium uptake protein, integral membrane component, KtrB      1
dtype: int64

PF13493.1
1957_A0A552YY26, FNJ58_01005, Sensor histidine kinase KdpD    111
4393_A0A2X0R081, kdpD, Sensor protein KdpD                     47
9370_G6F913, LLCRE1631_00006, Histidine kinase                  7

PF02669.10
1959_A0A0A7T690, kdpC, Potassium-transporting ATPase KdpC subunit     142
7433_G6F909, kdpC, Potassium-transporting ATPase KdpC subunit          15
8338_A0A0A7T690, kdpC, Potassium-transporting ATPase KdpC subunit      10
22051_A0A0A7T690, kdpC, Potassium-transporting ATPase KdpC subunit      1
22052_A0A0A7T690, kdpC, Potassium-transporting ATPase KdpC subunit      1
22050_A0A0A7T690, kdpC, Potassium-transporting ATPase KdpC subunit      1

PF03814.10
3291_A0A3N6L743, kdpA, Potassium-transporting ATPase potassium-binding subunit     88
5174_A0A4U1N4I2, kdpA, Potassium-transporting ATPase potassium-binding subunit     34
5173_A0A4U1N4I2, kdpA, Potassium-transporting ATPase potassium-binding subunit     28
6588_A0A1V0P1X6, kdpA, Potassium-transporting ATPase potassium-binding subunit     19
21875_A0A4U1N4I2, kdpA, Potassium-transporting ATPase potassium-binding subunit     1

PF02702.12
1957_A0A552YY26, FNJ58_01005, Sensor histidine kinase KdpD    111
4393_A0A2X0R081, kdpD, Sensor protein KdpD                     51
9370_G6F913, LLCRE1631_00006, Histidine kinase                  8

PF07274.7
853_A0A0B8QQW5, E34_1702, DUF1440 domain-containing protein        246
4213_A0A0A7T7J0, D4M07_10125, DUF1440 domain-containing protein     46

PF11361.3
837_U6ENZ7, BN927_02559, Uncharacterized protein    246
3308_T2F4S2, kw2_0761, Uncharacterized protein        1

PF01432.15
1770_Q9CEV7, pepF, Oligoendopeptidase F homolog                    187
2852_T0W231, LLT1_12110, Oligopeptidase PepB                       104
3982_T0WSF4, LLT3_09005, Oligopeptidase PepB                        63
4031_T0WSF4, LLT3_09005, Oligopeptidase PepB                        59
4234_A0A3N6KZX4, D6118_14265, Oligoendopeptidase F (Fragment)      43
10378_T0W231, LLT1_12110, Oligopeptidase PepB                        6
11367_A0A3N6KZX4, D6118_14265, Oligoendopeptidase F (Fragment)      5
13309_A0A3N6KZX4, D6118_14265, Oligoendopeptidase F (Fragment)      3
11375_T0UG73, LLT1_02400, Oligopeptidase PepB                        3
14976_A0A3N6KZX4, D6118_14265, Oligoendopeptidase F (Fragment)      2
14981_T0WSF4, LLT3_09005, Oligopeptidase PepB                        2
14980_A0A3N6KZX4, D6118_14265, Oligoendopeptidase F (Fragment)      2
18555_A0A3N6KZX4, D6118_14265, Oligoendopeptidase F (Fragment)      1
18551_A0A3N6KZX4, D6118_14265, Oligoendopeptidase F (Fragment)      1
18550_A0A3N6KZX4, D6118_14265, Oligoendopeptidase F (Fragment)      1
18549_A0A3N6KZX4, D6118_14265, Oligoendopeptidase F (Fragment)      1

PF07083.6
4078_A0A5D4G8H9, FYK05_04885, DUF1351 domain-containing protein     59
5959_G8P8D0, llh_10820, Uncharacterized protein                     25
6250_A0A4R5MQX0, C5L16_001028, Uncharacterized protein              22
7333_U6EMK2, BN927_02080, Uncharacterized protein                   15
9806_A0A0V8E0Y8, LMG9449_0693, Phage protein                         8
12386_A0A1P8BLY1, DS98104_11, Uncharacterized protein                4
13595_A0A418ZKA1, D4M07_05760, DUF1351 domain-containing protein     3
```

13063_A0A2Z3KHJ3, LL14B4_12765, Uncharacterized protein 2
15339_A0A1P8BLG1, DS98101_12, Uncharacterized protein 2
21535_A0A1B1IMV0, DS86501_11, Uncharacterized protein 1
20301_A0A199YP66, V425_10875, Uncharacterized protein 1
16090_A0A5D4G8H9, FYK05_04885, DUF1351 domain-containing protein 1
19432_A0A2Z3KE89, LL14B4_06080, Uncharacterized protein 1

PF12730.2
2721_A0A0V8EG05, E34_0809, ABC transporter permease 116
3693_A0A0V8EG05, E34_0809, ABC transporter permease 66
3667_A0A5D4GJ69, FYK05_02440, Lantibiotic ABC transporter permease 64
3670_Q48599, nisG, NisG 64
4151_A0A552YYN2, FNJ53_12290, ABC transporter permease 57
4150_A0A5E9J9T0, BU174_12365, ABC transporter permease 57
3327_A0A1V0PHD1, C5L16_002516, ABC transporter permease 46
7898_G6FG03, LLCRE1631_02446, Uncharacterized protein 12
8301_A0A1V0NUL7, LLJM4_1046, ABC transporter permease protein 11
9305_RXS50056, nan, hypothetical protein 9
8860_D2BQU2, LLKF_1218, ABC transporter, permease protein 6
11590_A0A0V8DPV6, LMG9449_2213, ABC transporter permease protein 5
10061_A0A0V8EGA7, N42_2256, Putative transporter trans-membrane domain bacteriocin immunity protein 5
11363_G0WJM2, smbT, PreSmb transportation protein 5
11139_Q9RAU9, lacG, LacG 5
10060_T0VBR6, LLT3_00175, Uncharacterized protein 5
12037_Q9RAU9, lacG, LacG 4
13350_O34115, lctG, LctG 3
13351_O34114, lctE, LctE 3
13734_A0A552YYN2, FNJ53_12290, ABC transporter permease 3
13733_A0A5E9J9T0, BU174_12365, ABC transporter permease 3
7565_A0A1V0NUQ4, LLCC_2147, ABC transporter permease protein 1
1619_A0A552YMR9, FNJ58_11205, ABC transporter permease 1
22549_A0A098CZD1, LGMT14_02210, Transport permease protein 1

PF12730.2
2721_A0A0V8EG05, E34_0809, ABC transporter permease 116
3693_A0A0V8EG05, E34_0809, ABC transporter permease 66
3667_A0A5D4GJ69, FYK05_02440, Lantibiotic ABC transporter permease 64
3670_Q48599, nisG, NisG 64
4151_A0A552YYN2, FNJ53_12290, ABC transporter permease 57
4150_A0A5E9J9T0, BU174_12365, ABC transporter permease 57
3327_A0A1V0PHD1, C5L16_002516, ABC transporter permease 46
7898_G6FG03, LLCRE1631_02446, Uncharacterized protein 12
8301_A0A1V0NUL7, LLJM4_1046, ABC transporter permease protein 11
9305_RXS50056, nan, hypothetical protein 9
8860_D2BQU2, LLKF_1218, ABC transporter, permease protein 6
11590_A0A0V8DPV6, LMG9449_2213, ABC transporter permease protein 5
10061_A0A0V8EGA7, N42_2256, Putative transporter trans-membrane domain bacteriocin immunity protein 5
11363_G0WJM2, smbT, PreSmb transportation protein 5
11139_Q9RAU9, lacG, LacG 5
10060_T0VBR6, LLT3_00175, Uncharacterized protein 5
12037_Q9RAU9, lacG, LacG 4
13350_O34115, lctG, LctG 3
13351_O34114, lctE, LctE 3
13734_A0A552YYN2, FNJ53_12290, ABC transporter permease 3
13733_A0A5E9J9T0, BU174_12365, ABC transporter permease 3
7565_A0A1V0NUQ4, LLCC_2147, ABC transporter permease protein 1
1619_A0A552YMR9, FNJ58_11205, ABC transporter permease 1
22549_A0A098CZD1, LGMT14_02210, Transport permease protein 1

PF05649.8
685_A0A552YTN0, pepO, Endopeptidase PepO 176
2313_G0WJP0, orf33, Oligoendopeptidase O 105
6385_A0A1V0PIK6, C5L16_000247, Oligoendopeptidase O 18
5690_A0A1V0PDD4, LLJM1_MP0164, Oligoendopeptidase O 13
2045_A0A552YGA9, FNJ53_13730, Peptidase M13 (Fragment) 8
11041_T0TDV5, LLT6_15115, Peptidase M13 4
11035_G0WJP0, orf33, Oligoendopeptidase O 3
14190_A0A1V0PDD4, LLJM1_MP0164, Oligoendopeptidase O 1
9154_A0A0B8QME1, JCM5805K_2315, Predicted metalloendopeptidase 1

PF02486.14
3424_T0VDW8, LLT3_10780, Replication initiation protein 75
3634_A0A2A9ISH2, BW154_01510, Transcriptional regulator 74
3600_D2BN15, LLKF_0307, Transcriptional regulator, Cro/CI family 70
4557_A0A5D4G3I1, FYK05_08965, Replication initiation factor domain-containing protein 46
4715_G6FEC2, LLCRE1631_01865, HTH cro/C1-type domain-containing protein 41
6230_A0A1V0P0E9, LLUC06_0643, Phosphoglucomutase 21
6375_Q2VHS6, orf4, Putative replication initiation factor 19
6863_A0A1V0NZV7, LLUC06_0361, Transcriptional regulator 18
7255_A0A4V2EOI5, EQJ87_03990, Replication initiation protein 17
7304_T0WQZ9, LLT3_02030, Uncharacterized protein 13
8688_A0A1V0NZV7, LLUC06_0361, Transcriptional regulator 11
8493_G8PA44, llh_14170, Putative replication initiation factor 10
8377_A0A1V0PFQ2, C5L16_000944, Transcriptional regulator, Cro/CI family 10
9107_D2BMA3, LLKF_2243, Replication initiation factor 9
9476_A0A2X0QYV8, AMHIJAGA_01004, Uncharacterized protein 7
9391_A0A1E7G1U0, AJ89_13205, Transcriptional regulator 7

```
10392_A0A2X0SKP3, AMHIJAGA_01013, Rol_Rep_N domain-containing protein                    6
9991_A0A2X0SKP3, AMHIJAGA_01013, Rol_Rep_N domain-containing protein                     6
11488_A0A1E7G0I8, AJ89_13775, Cro/Cl family transcriptional regulator                    5
11906_A0A2X0QYV8, AMHIJAGA_01004, Uncharacterized protein                                4
13518_no_reference_sequence, nan, hypothetical protein                                   3
11090_A0A2A5SJF4, RU90_GL001998, Transcriptional regulator, Cro/CI family                3
13869_A0A2A9ISH2, BW154_01510, Transcriptional regulator                                 3
15730_A0A084ABP4, U725_01116, Cro/Cl family transcriptional regulator                    2
12988_Q2VHS6, orf4, Putative replication initiation factor                               2
19885_O32790, repD, RepD protein                                                         1
19395_A0A0V8CKI1, KF282_2515, Transcriptional regulator Cro/CI family                    1
22744_A0A3N6KY75, D6118_05875, XRE family transcriptional regulator                      1
18686_no_reference_sequence, nan, hypothetical protein                                   1
18673_T0WSY5, LLT3_13775, Uncharacterized protein (Fragment)                             1
18873_A0A2X0QYV8, AMHIJAGA_01004, Uncharacterized protein                                1


PF04103.10
6622_A0A552YRV6, FNJ58_07630, Uncharacterized protein              19
11249_A0A552XIB5, FNJ55_06855, DUF4190 domain-containing protein     4


PF04967.7
4874_T0V290, LLT1_13610, Uncharacterized protein                                                                   6
4361_Q04379, nan, L.lactis plasmid pSL2 replication region (ClaI-PvuI) DNA for putative replication protein    5
6433_O54684, nan, HTH arsR-type domain-containing protein                                                          3
7569_A0A1V0PDX7, LLJM1_pE07, RepB family protein                                                                   1
6317_G6FG90, LLCRE1631_02533, Uncharacterized protein                                                              1
13272_T0SAH5, LLT6_03400, Uncharacterized protein (Fragment)                                                       1
1934_Q7DH39, pLd9_2, Uncharacterized protein                                                                       1
9247_T0VID9, LLT3_00155, RepB family protein                                                                       1
11704_A0A084ADA2, U725_00534, Uncharacterized protein                                                              1
13842_Q4LEN6, nan, Uncharacterized protein                                                                         1
18814_T0SAH5, LLT6_03400, Uncharacterized protein (Fragment)                                                       1


PF02796.10
3148_G0WJP3, orf30, Site-specific recombinase                                                                 99
3156_K4G131, nan, Site-specific recombinase                                                                   97
2135_A0A1V0NX14, pinR, DNA invertase                                                                          54
3949_A0A098CZA4, hin_1, DNA-invertase hin                                                                     49
4458_A0A552Z1N1, FNJ53_14105, Recombinase family protein                                                      47
4979_A0A4U1MYN0, E6O52_11720, Recombinase family protein                                                      30
8126_A0A552Z1N1, FNJ53_14105, Recombinase family protein                                                      19
6774_K4G131, nan, Site-specific recombinase                                                                   19
6955_A0A552YH52, FNJ58_14035, Recombinase family protein                                                      18
6273_K4G131, nan, Site-specific recombinase                                                                   17
7334_K4G131, nan, Site-specific recombinase                                                                   16
7894_A0A098CZA4, hin_1, DNA-invertase hin                                                                     12
8110_A0A5D4GT25, FYK05_00070, Helix-turn-helix domain-containing protein (Fragment)                           12
7684_A0A0D4CCF0, nan, Resolvase                                                                               11
8421_A0A2Z3KH92, LL14B4_03190, Resolvase                                                                      11
8419_K4G131, nan, Site-specific recombinase                                                                   11
9165_A0A098CZA4, hin_1, DNA-invertase hin                                                                      9
9533_G0WJP3, orf30, Site-specific recombinase                                                                  8
7903_A0A1V0NLP6, LLUC11_0642, Resolvase                                                                        8
9972_A0A4U1MYN0, E6O52_11720, Recombinase family protein                                                       7
8802_K4G131, nan, Site-specific recombinase                                                                    7
8438_A0A0H1RIT1, VN91_2610, Transposon DNA-invertase                                                           7
9961_G0WJP3, orf30, Site-specific recombinase                                                                  7
10448_A0A552YH52, FNJ58_14035, Recombinase family protein                                                      6
11064_G0WJP3, orf30, Site-specific recombinase                                                                 5
11061_A0A098CY12, hin_2, DNA-invertase hin                                                                     4
11951_K4G131, nan, Site-specific recombinase                                                                   4
11947_K4G131, nan, Site-specific recombinase                                                                   4
11270_A0A0H1RQJ7, VN91_0505, Transposon Tn1546 resolvase                                                       4
12645_A0A3B0GFY4, D8M10_09985, Helix-turn-helix domain-containing protein                                      4
11074_A0A0H1RIT1, VN91_2610, Transposon DNA-invertase                                                          3
13025_A0A552YH52, FNJ58_14035, Recombinase family protein                                                      3
13046_A0A4U1MYN0, E6O52_11720, Recombinase family protein                                                      3
13023_A0A098CZA4, hin_1, DNA-invertase hin                                                                     2
13027_T0W9D4, LLT1_01405, Resolvase/invertase-type recombinase catalytic domain-containing protein             2
14200_A0A552X906, FNJ55_14345, Recombinase family protein                                                      2
16804_K4G131, nan, Site-specific recombinase                                                                   1
16814_A0A552Z1N1, FNJ53_14105, Recombinase family protein                                                      1
20534_A0A5C8JGG7, FVP42_11265, Recombinase family protein                                                      1
16810_A0A552YH52, FNJ58_14035, Recombinase family protein                                                      1
16813_A0A0M2ZYY4, VN96_0422, CRISPR locus-like DNA-binding protein                                             1
16799_K4G131, nan, Site-specific recombinase                                                                   1
20312_A0A098CZA4, hin_1, DNA-invertase hin                                                                     1
16777_A0A098CZA4, hin_1, DNA-invertase hin                                                                     1
16802_K4G131, nan, Site-specific recombinase                                                                   1
14238_A0A552YH52, FNJ58_14035, Recombinase family protein                                                      1
14222_A0A098CZA4, hin_1, DNA-invertase hin                                                                     1
14202_K4G131, nan, Site-specific recombinase                                                                   1
16801_G0WJP3, orf30, Site-specific recombinase                                                                 1
16776_A0A1E7G0E7, AJ89_14420, Resolvase                                                                        1
16818_A0A199YR78, V425_09490, Transposon Tn552 DNA-invertase BinR                                              1
```
