# Supplementary material for: Machine learning predicts and provides insights into milk acidification rates of Lactococcus lactis
Source: PLoS One. 2021 Mar 15;16(3):e0246287. doi: 10.1371/journal.pone.0246287 (PMC7959382; doi:10.1371/journal.pone.0246287)
Supplement: S2 File — (PDF) [file pone.0246287.s006.pdf]

The 9-mers with the highest feature importances and the genes in which they occur

```
AGGGCCCAG / CTGGGCCCT
763_G0WJQ2, lacG, 6-phospho-beta-galactosidase                          281
1933_G6F953, LLCRE1631_00046, Uncharacterized protein                    16
1432_Q9CHW5, serC, Phosphoserine aminotransferase                         4
16061_A0A1V0PDW7, LLJM1_pD08, Type IIS restriction endonuclease           1
11710_A0A1V0PDW2, LLJM1_pD07, Type IIS restriction/modification enzyme    1
14758_GCF_002148215.1_ASM214821v1_genomic, nan, hypothetical protein      1
```

```
CCGAGACCG / CGGTCTCGG
763_G0WJQ2, lacG, 6-phospho-beta-galactosidase                     281
1459_A0A1V0NE97, FYK05_02750, Tim44 domain-containing protein     224
11727_A0A1V0NE97, FYK05_02750, Tim44 domain-containing protein      2
```

```
AAGATCTAC / GTAGATCTT
718_A0A5E9JHB6, BU174_06410, Glutamate synthase large subunit                                          229
720_A0A0B8QZB7, yhaM, 3'-5' exoribonuclease YhaM                                                       190
1787_A0A0A7T031, FYK05_06930, Cytochrome c-type biosis protein DsbD protein-disulfide reductase        182
1957_A0A552YY26, FNJ58_01005, Sensor histidine kinase KdpD                                             110
3164_A0A552XG60, FNJ55_08750, Helix-turn-helix transcriptional regulator                                86
4393_A0A2X0R081, kdpD, Sensor protein KdpD                                                              46
4619_A0A0B8QZB7, yhaM, 3'-5' exoribonuclease YhaM                                                       40
5054_A2RKX4, telC, Putative tellurium resistance protein                                                35
4605_A0A1V0P2E0, LLUC06_1421, Metallophos domain-containing protein                                     18
6818_A0A1V0P3T2, LLUC06_1890, Prophage protein                                                          18
3822_A2RLN2, llmg_1628, Putative secreted protein                                                       17
5961_A0A552XJP5, FNJ55_05160, DUF4767 domain-containing protein                                         15
7300_A0A1V0PDT6, LLJM1_pC10, DNA restrictase, restriction system type III                               14
2027_GCF_000014545.1_ASM1454v1_genomic, nan, hypothetical protein                                       10
2167_T0W5N0, metE, 5-methyltetrahydropteroyltriglutamate--homocysteine methyltransferase                10
1232_Q9CE01, pepX, Xaa-Pro dipeptidyl-peptidase                                                          8
9370_G6F913, LLCRE1631_00006, Histidine kinase                                                           7
9530_A0A0V8E467, M20_1414, Glycosyltransferase                                                           6
10183_A0A5D4GGJ8, FYK05_06720, Uncharacterized protein                                                   6
8862_D2BQU4, npkS, Hybrid nonribosomal peptide synthetase/polyketide synthase                            6
9515_A0A552YXF7, FNJ53_12860, Restriction endonuclease subunit S                                         5
12568_A0A2A9IR26, BW154_10055, McbB family protein                                                       4
12629_Q02YN3, LACR_1424, Type I restriction-modification system methyltransferase subunit                4
11028_no_reference_sequence, nan, hypothetical protein                                                   4
9409_A0A0V8E4G2, LMG9449_0570, Prophage ps1 protein 11                                                   4
1678_G6FG47, LLCRE1631_02490, Uncharacterized protein                                                    4
13625_no_reference_sequence, nan, hypothetical protein                                                   3
11276_A0A0V8E7U2, M20_0717, Uncharacterized protein                                                      3
3289_Q9AZS9, orf43, Portal protein                                                                       3
13488_no_reference_sequence, nan, hypothetical protein                                                   3
4115_Q7DH38, hsdS, Type I R/M system specificity subunit                                                 3
15277_A0A418ZNG0, D4M07_01510, XRE family transcriptional regulator                                      2
14291_no_reference_sequence, nan, hypothetical protein                                                   2
15102_no_reference_sequence, nan, hypothetical protein                                                   2
10513_Q8GRC4, hsdS, Type I R/M system specificity subunit                                                2
6143_Q02ZU2, LACR_0988, DUF4767 domain-containing protein                                                2
15402_no_reference_sequence, nan, hypothetical protein                                                   2
15236_A0A552YX59, FNJ58_01435, NAD-dependent epimerase/dehydratase family protein                        2
16367_A0A0M2ZQE9, VN96_1798, Serine/threonine protein phosphatase                                        1
18599_A0A2A5RL87, RU85_GL000973, AlwI restriction endonuclease                                           1
6488_T0UC80, LLT7_14850, Uncharacterized protein                                                         1
12965_A0A0V8BKJ3, LKF67_1710, Type I restriction-modification system specificity subunit S               1
6484_U6EQ36, BN927_00037, Type I restriction-modification system,specificity subunit S                   1
21772_Q9AZJ2, orf17, Orf17                                                                               1
9933_G6FG91, LLCRE1631_02534, Type I site-specific deoxyribonuclease                                     1
20178_no_reference_sequence, nan, hypothetical protein                                                   1
7351_A0A1V0P117, LLUC06_0958, Prophage TAL                                                               1
3714_A0A3N6N586, D6118_07250, Metallophosphoesterase                                                     1
3172_H2AM39, hsdS, Putative methylase S                                                                  1
19861_A0A0V8CX02, LMG8520_2307, Phage N-acetylmuramoyl-L-alanine amidase                                 1
1130_A0A0B8R0U6, acpS, Holo-[acyl-carrier-protein] synthase                                              1
6223_A0A5D4GE92, FYK05_04305, Phage portal protein                                                       1
12307_A0A2A5SNV9, RU92_GL001671, Restriction endonuclease                                                1
```

```
GCCGTCGAC / GTCGACGGC
1963_A0A2X0PDC6, prtP, PIII-type proteinase                                                 132
378_H5SYE2, gyrB, DNA gyrase subunit B                                                       35
8980_A0A2N5WBV3, rstR_2, Cryptic phage CTXphi transcriptional repressor RstR                 10
6105_A0A4U1N3H8, E6O52_04885, Uncharacterized protein                                         8
9899_A0A3N6KLP2, D6118_13445, LPXTG cell wall anchor domain-containing protein                6
9106_A0A4R5N401, C5L16_000978, GRAM_POS_ANCHORING domain-containing protein                   4
622_A0A4U1N0I6, E6O52_13445, Multidrug efflux MFS transporter                                 1
22710_A0A2X0PDC6, prtP, PIII-type proteinase                                                  1
22691_A0A0M2ZP19, VN96_2651, Type VII secretion-associated serine protease mycosin, mycP      1
14064_T0WSZ7, LLT3_01170, Peptidase S8                                                        1
16392_A0A2X0PDC6, prtP, PIII-type proteinase                                                  1
```

AGAGTCCGG / CCGGACTCT

756_P23531, lacE, PTS system lactose-specific EIICB component	280
3516_T2F7S7, kw2_1739, Cell surface protein	18
8981_A0A2A9IQ55, BW151_05360, Uncharacterized protein	10
4012_A2RKM7, arsA, Arsenical pump-driving ATPase	8
11235_A0A443L7J1, EO246_10615, Glycoside hydrolase, family 25	4
3328_A0A5E9JJ36, BU174_07780, Serine protease	2
19564_no_reference_sequence, nan, hypothetical protein	1

CCGGGTAGC / GCTACCCGG

1963_A0A2X0PDC6, prtP, PIII-type proteinase	141
9899_A0A3N6KLP2, D6118_13445, LPXTG cell wall anchor domain-containing protein	6
1543_A0A1V0NCX9, LL275_0137, ABC transporter permease protein	3
9106_A0A4R5N401, C5L16_000978, GRAM_POS_ANCHORING domain-containing protein	3
12934_P16271, prtP, PI-type proteinase	2
15258_A0A224X4G4, RsY01_1176, N6_N4_Mtase domain-containing protein	2
14064_T0WSZ7, LLT3_01170, Peptidase S8	2
15349_A0A098CZN7, rmlA1_2, Glucose-1-phosphate thymidylyltransferase 1	2
16396_Q7M177, nan, Lactocepin (Fragments)	1
19357_no_reference_sequence, nan, hypothetical protein	1
16392_A0A2X0PDC6, prtP, PIII-type proteinase	1
6213_A0A552XBB2, FNJ55_13240, DUF389 domain-containing protein	1

GCCAGGGAC / GTCCCTGGC

1963_A0A2X0PDC6, prtP, PIII-type proteinase	137
5684_A2RL04, traD, Conjugal transfer protein TraD	15
9290_T0S4R4, LLT6_13070, Uncharacterized protein	9
9899_A0A3N6KLP2, D6118_13445, LPXTG cell wall anchor domain-containing protein	6
9106_A0A4R5N401, C5L16_000978, GRAM_POS_ANCHORING domain-containing protein	3
8127_A0A1V0NBY2, LL275_pA091, Recombinase/resolvase/invertase	3
811_H5SXV8, floL, Flotillin-like protein	3
5570_A0A0V8EQI8, N42_0733, GRAM_POS_ANCHORING domain-containing protein	3
9678_A0A4R5N3T8, C5L16_001374, Uncharacterized protein	2
686_A0A0B8QRZ5, JCM5805K_2525, Carbon starvation protein A	2
22700_Q7M177, nan, Lactocepin (Fragments)	1
19125_A0A2X0STZ2, AMHIJAGA_01418, Uncharacterized protein	1
22693_A0A1V0PDD3, LLJM1_MP0161, Lactoceptin PrtP	1
22699_Q7M177, nan, Lactocepin (Fragments)	1
16395_A0A2X0PDC6, prtP, PIII-type proteinase	1
14064_T0WSZ7, LLT3_01170, Peptidase S8	1
16392_A0A2X0PDC6, prtP, PIII-type proteinase	1
1753_A0A552YRJ7, FNJ58_07930, GRAM_POS_ANCHORING domain-containing protein	1
22708_P16271, prtP, PI-type proteinase	1
16396_Q7M177, nan, Lactocepin (Fragments)	1

CTACCCGGC / GCCGGGTAG

1963_A0A2X0PDC6, prtP, PIII-type proteinase	141
9899_A0A3N6KLP2, D6118_13445, LPXTG cell wall anchor domain-containing protein	6
9106_A0A4R5N401, C5L16_000978, GRAM_POS_ANCHORING domain-containing protein	3
12934_P16271, prtP, PI-type proteinase	2
14064_T0WSZ7, LLT3_01170, Peptidase S8	2
16396_Q7M177, nan, Lactocepin (Fragments)	1
16392_A0A2X0PDC6, prtP, PIII-type proteinase	1
14082_A0A4Q7DLN6, EQJ87_11380, Uncharacterized protein	1
12945_A0A1V0NN90, LLUC11_1145, Uncharacterized protein	1

CGCGGCGTA / TACGCCGCG

1963_A0A2X0PDC6, prtP, PIII-type proteinase	138
9899_A0A3N6KLP2, D6118_13445, LPXTG cell wall anchor domain-containing protein	6
12934_P16271, prtP, PI-type proteinase	3
2765_T0WUH0, LLT3_06515, Multidrug ABC transporter ATP-binding protein	2
19803_A0A343JPS6, 57001_11, Neck passage structure	1
16395_A0A2X0PDC6, prtP, PIII-type proteinase	1
22717_Q9AIQ2, prtP, PrtP	1
9106_A0A4R5N401, C5L16_000978, GRAM_POS_ANCHORING domain-containing protein	1
22691_A0A0M2ZP19, VN96_2651, Type VII secretion-associated serine protease mycosin, mycP	1
11105_A0A343JPS6, 57001_11, Neck passage structure	1
20052_A0A343JPS6, 57001_11, Neck passage structure	1
14064_T0WSZ7, LLT3_01170, Peptidase S8	1
16392_A0A2X0PDC6, prtP, PIII-type proteinase	1

CGCCGCGGC / GCCGCGGCG

1963_A0A2X0PDC6, prtP, PIII-type proteinase	139
2633_A2RLX5, copA, Copper/potassium-transporting ATPase	111
9899_A0A3N6KLP2, D6118_13445, LPXTG cell wall anchor domain-containing protein	6
9106_A0A4R5N401, C5L16_000978, GRAM_POS_ANCHORING domain-containing protein	3
12934_P16271, prtP, PI-type proteinase	2
14064_T0WSZ7, LLT3_01170, Peptidase S8	2
22693_A0A1V0PDD3, LLJM1_MP0161, Lactoceptin PrtP	1
16396_Q7M177, nan, Lactocepin (Fragments)	1
19491_A0A552YP00, FNJ58_09685, Uncharacterized protein	1
16392_A0A2X0PDC6, prtP, PIII-type proteinase	1

CGCAGCCCC / GGGGCTGCG

614_A0A0A7SYJ0, ytoI, CBS domain-containing protein	107

457_A0A0A7SWZ7, D4M07_07450, HAD family hydrolase	78
3769_Q9CH23, tagZ, Teichoic acid biosynthesis protein	68
3885_Q9CGA7, citF, Citrate lyase alpha chain	62
1914_A0A0V8EKC4, leuB, 3-isopropylmalate dehydrogenase	57
3452_Q9CH23, tagZ, Teichoic acid biosynthesis protein	57
3901_A0A5E9JIP8, argC, N-acetyl-gamma-glutamyl-phosphate reductase	56
3209_A2RHW4, llmg_0246, NAD(P)-bd_dom domain-containing protein	34
599_G6FCB7, LLCRE1631_01160, MFS domain-containing protein	33
6332_G8P623, argC, N-acetyl-gamma-glutamyl-phosphate reductase	11
3224_A0A3N6LC00, leuB, 3-isopropylmalate dehydrogenase	4
9079_A0A0A7SYJ0, ytoI, CBS domain-containing protein	3
16153_A0A5E9JIP8, argC, N-acetyl-gamma-glutamyl-phosphate reductase	2
20552_A0A0V8EPM4, N42_1004, Putative CDP-glycosylpolyol phosphate:glycosylpolyol glycosylpolyolphosphotransferase	1
22241_A0A0V8EPM4, N42_1004, Putative CDP-glycosylpolyol phosphate:glycosylpolyol glycosylpolyolphosphotransferase	1
16281_Q9CH23, tagZ, Teichoic acid biosynthesis protein	1
13913_T0S6L0, argC, N-acetyl-gamma-glutamyl-phosphate reductase	1
22423_G8P6I3, llh_9195, Uncharacterized protein	1
22422_G8P6I3, llh_9195, Uncharacterized protein	1
11952_A0A552YWL0, FNJ58_01190, MFS transporter	1
16152_T0S6L0, argC, N-acetyl-gamma-glutamyl-phosphate reductase	1

ACTGGGCCC / GGGCCCAGT

763_G0WJQ2, lacG, 6-phospho-beta-galactosidase	281
2822_T0VIZ6, LLT3_03140, Glyoxalase	106
1073_A0A0B8QXM0, FYK05_10510, Glyoxalase family protein	50
10647_A0A552XDM7, FNJ55_11285, Glycosyltransferase family 4 protein	6
1432_Q9CHW5, serC, Phosphoserine aminotransferase	4
3832_A0A5D4GFQ0, FYK05_05065, Phage tail tape measure protein	1
4612_Q02ZE2, LACR_1146, Minor tail protein gp26-like protein	1
16061_A0A1V0PDW7, LLJM1_pD08, Type IIS restriction endonuclease	1
11710_A0A1V0PDW2, LLJM1_pD07, Type IIS restriction/modification enzyme	1
14758_GCF_002148215.1_ASM214821v1_genomic, nan, hypothetical protein	1

GATCTAACC / GGTTAGATC

2374_A0A1E7G582, AJ89_04985, Cation transporter	112
3148_G0WJP3, orf30, Site-specific recombinase	92
4005_B7SFX6, pyrF_1, Orotidine 5'-phosphate decarboxylase	60
3949_A0A098CZA4, hin_1, DNA-invertase hin	43
5104_A0A1E7G225, AJ89_10755, Acyl_transf_3 domain-containing protein	29
5475_A0A552YKS5, FNJ58_11750, Site-specific integrase	26
3156_K4G131, nan, Site-specific recombinase	24
6741_A0A1V0P056, pyrF, Orotidine 5'-phosphate decarboxylase	19
6955_A0A552YH52, FNJ58_14035, Recombinase family protein	17
7764_A0A1E7G0A0, AJ89_14585, Uncharacterized protein	14
8204_A0A3N6KUH8, D6118_08800, Uncharacterized protein	12
8220_A0A3N6MWZ8, D6118_08885, Uncharacterized protein	12
8619_A0A084A9S6, U725_01810, Ser/Thr protein Kinase	11
8910_A0A199YQN0, V425_11060, Uncharacterized protein	10
8126_A0A552Z1N1, FNJ53_14105, Recombinase family protein	9
4979_A0A4U1MYN0, E6052_11720, Recombinase family protein	9
9701_A0A0D6E075, LACPI_2408, Peptidase C51 domain-containing protein	8
5512_A0A451F063, nan, UDP-glucose 6-dehydrogenase	7
9533_G0WJP3, orf30, Site-specific recombinase	7
9165_A0A098CZA4, hin_1, DNA-invertase hin	6
2361_A0A1V0P8B2, dnaG, DNA primase	6
10062_A0A0V8EGR7, N42_2255, ABC-type multidrug transport system ATPase component	5
9961_G0WJP3, orf30, Site-specific recombinase	5
11064_G0WJP3, orf30, Site-specific recombinase	4
2859_A0A1V0NK65, LLUC11_0135, GRAM_POS_ANCHORING domain-containing protein	4
10448_A0A552YH52, FNJ58_14035, Recombinase family protein	4
7684_A0A0D4CCF0, nan, Resolvase	4
12744_U6ENV9, BN927_02475, Phage tail fibers	4
9972_A0A4U1MYN0, E6052_11720, Recombinase family protein	2
6273_K4G131, nan, Site-specific recombinase	2
	...
22152_S6EZI1, int, Integrase	1
16810_A0A552YH52, FNJ58_14035, Recombinase family protein	1
7334_K4G131, nan, Site-specific recombinase	1
11947_K4G131, nan, Site-specific recombinase	1
18598_no_reference_sequence, nan, hypothetical protein	1
16818_A0A199YR78, V425_09490, Transposon Tn552 DNA-invertase BinR	1
8802_K4G131, nan, Site-specific recombinase	1
16815_A0A3N6KLK1, D6118_13800, Recombinase family protein	1
16530_A0A1E7G232, AJ89_10750, Acyl_transf_3 domain-containing protein	1
11074_A0A0H1RIT1, VN91_2610, Transposon DNA-invertase	1
4757_no_reference_sequence, nan, hypothetical protein	1
16533_A0A1E7G232, AJ89_10750, Acyl_transf_3 domain-containing protein	1
16532_A0A1E7G232, AJ89_10750, Acyl_transf_3 domain-containing protein	1
16801_G0WJP3, orf30, Site-specific recombinase	1
13027_T0W9D4, LLT1_01405, Resolvase/invertase-type recombinase catalytic domain-containing protein	1
18506_no_reference_sequence, nan, hypothetical protein	1
18956_A0A3S4MEU3, EO246_00770, XRE family transcriptional regulator	1
6719_A0A3N6L7S0, AMHIJAGA_01412, Site-specific integrase	1
16531_A0A1E7G232, AJ89_10750, Acyl_transf_3 domain-containing protein	1
14238_A0A552YH52, FNJ58_14035, Recombinase family protein	1
14200_A0A552X906, FNJ55_14345, Recombinase family protein	1
16534_A0A1E7G232, AJ89_10750, Acyl_transf_3 domain-containing protein	1
19845_A0A0V8BYZ0, LKF67_0683, Putative ATP-dependent endonuclease OLD family	1
11730_G6FG94, LLCRE1631_02537, Uncharacterized protein	1

```
10282_G6FG94, LLCRE1631_02537, Uncharacterized protein                                             1
4458_A0A552Z1N1, FNJ53_14105, Recombinase family protein                                           1
8318_D2BPF9, LLKF_0693, Integrase/recombinase                                                      1
10300_T2DII5, muc, Mucus-binding protein                                                           1
16794_A0A098CY12, hin_2, DNA-invertase hin                                                         1
16778_A0A3N6KLK1, D6118_13800, Recombinase family protein                                          1


CGGAACCCG / CGGGTTCCG
2349_A0A1E7G6T4, AJ89_02035, Diphosphomevalonate decarboxylase                                  112
725_H5SXG2, ygdA, Ribosome hibernation promoting factor                                            3
10995_A0A4U1N3P2, E6O52_02920, Type IV secretory system conjugative DNA transfer family protein      1
11066_A0A1V0NLG8, LLUC11_0585, Abortive infection protein                                          1


CGACCTACA / TGTAGGTCG
1798_U6ERA5, BN927_01503, Potassium uptake protein, integral membrane component, KtrB     184
689_T0SFM9, LLT6_11740, Molecular chaperone GroES                                          84
2927_A2RIU1, kup2, Probable potassium transport system protein kup 2                       82
855_Q9CHU4, kup2, Probable potassium transport system protein kup 2                        30
6170_A0A2X0R671, AMHIJAGA_03057, Uncharacterized protein                                   24
6165_A0A2X0SR36, AMHIJAGA_03059, Prophage pi3 protein 47, replisome organiser              18
6958_A0A5D4G9W6, FYK05_06675, Single-stranded DNA-binding protein                          18
6225_A0A552XIA1, FNJ55_08010, Zinc-dependent alcohol dehydrogenase family protein          14
9289_A0A0V8BJY6, LKF67_1738, Alpha-D-GlcNAc alpha-12-L-rhamnosyltransferase                 9
641_A0A2A9I5E7, BW151_04910, ABC transporter                                                6
4936_A0A3Q9TCW2, nan, Glycosyltransferase                                                   6
11602_A0A2X0R8N9, AMHIJAGA_01393, DUF1972 domain-containing protein                         5
214_A0A0V8DC55, asd, Aspartate-semialdehyde dehydrogenase                                   4
823_G8P569, llh_8075, Uncharacterized protein                                               3
14146_U6ERA5, BN927_01503, Potassium uptake protein, integral membrane component, KtrB      1


AGCCCTATG / CATAGGGCT
1879_A0A199YU45, V425_04615, Uncharacterized protein                           172
3068_A0A4R5MRI2, C5L16_001022, Uncharacterized protein                          94
4068_A0A1V0ND03, LL275_0166, DNA-binding helix-turn-helix protein               56
3735_A0A1V0P1M8, LLUC06_1164, Uncharacterized protein                           55
4304_T0VG54, LLT3_12415, AMP-binding protein                                    49
5251_A0A0M2ZRS8, tagH, Teichoic acids export ATP-binding protein TagH           34
4881_Q9AZP3, orf19, Orf19                                                       23
5385_A0A3N6KZ20, D6118_06795, DUF658 domain-containing protein                  21
5893_K4EG45, pLP712_27, Uncharacterized protein                                 12
7717_A0A0V8AUD8, E34_0209, Glycosyltransferase RgpE                             11
319_Q9CJ54, tgt, Queuine tRNA-ribosyltransferase                                10
6073_S6ET33, lr2004, Rep_3 domain-containing protein                             1
13785_Q9AZP3, orf19, Orf19                                                       1
19581_A0A1B1IMP4, DS62501_47, Putative receptor binding protein                  1
19168_no_reference_sequence, nan, ATP-dependent DNA helicase Rep                 1
12444_no_reference_sequence, nan, Mannose-1-phosphate guanylyltransferase 1      1


CACGCTGCG / CGCAGCGTG
766_P23496, lacX, Protein LacX, plasmid                                         281
2052_T0VLT5, LLT1_02685, MFS transporter permease                               138
96_A0A3N6L4H6, D6118_09825, ABC transporter ATP-binding protein                  77
5492_A0A1V0PC36, LLJM3_2146, Helicase                                            16
8548_A0A3N6MUB6, D6118_10420, Uncharacterized protein                            11
11809_T0VLT5, LLT1_02685, MFS transporter permease                                4
3957_A0A1B1RSL6, FNJ55_13995, ATP-dependent Clp protease ATP-binding subunit      4
12319_A0A5E9J974, BU174_12355, Phage_integrase domain-containing protein          4
10916_A0A2Z5ZA93, LLCC_2854, Major facilitator superfamily permease               3
12149_GCF_002078855.1_ASM207885v1_genomic, nan, hypothetical protein              3
```
