# Supplementary material for: Machine learning predicts and provides insights into milk acidification rates of Lactococcus lactis
Source: PLoS One. 2021 Mar 15;16(3):e0246287. doi: 10.1371/journal.pone.0246287 (PMC7959382; doi:10.1371/journal.pone.0246287)
Supplement: S3 File — (PDF) [file pone.0246287.s007.pdf]

The 8-mers with the highest feature importances and the genes in which they occur

AACCGGGG / CCCCGGTT

1093_Q9CIE4, ligA, DNA ligase	229
1569_A0A1V0NFJ4, FYK05_05295, Uncharacterized protein	218
1605_A0A1V0NGV3, FYK05_03555, Alpha-1,2-mannosidase	211
271_A0A552YLZ9, FNJ58_10890, Carboxylate--amine ligase	143
1462_A0A1V0P2J4, LLUC06_1484, Uncharacterized protein	142
1955_Q07741, oppA, Oligopeptide-binding protein OppA	128
3379_A0A552YVN8, FNJ53_13720, Oligopeptide ABC transporter substrate-binding protein	63
3621_Q9CGA2, ymbJ, Uncharacterized protein	63
599_G6FCB7, LLCRE1631_01160, MFS domain-containing protein	46
4149_A0A552XPH1, spxB, Pyruvate oxidase	41
4606_Q9AZL2, orf53, Host-specificity	34
5378_G8P9N3, llh_11870, Pyruvate oxidase	32
5441_A0A1E7G583, AJ89_04505, Sugar ABC transporter permease	25
6413_T0WSF2, LLT3_08960, Peptide-binding protein	20
5077_A0A2Z5Z723, leuC, 3-isopropylmalate dehydratase large subunit	15
6414_A0A552YVN8, FNJ53_13720, Oligopeptide ABC transporter substrate-binding protein	13
3586_A2RIG2, llmg_0453, Similar to sucrose-specific PTS enzyme IIABC	11
7721_G8P4I3, llh_1250, Transport permease protein	11
7934_A0A0M2ZQE9, VN96_1798, Serine/threonine protein phosphatase	10
8628_A0A552YVN8, FNJ53_13720, Oligopeptide ABC transporter substrate-binding protein	10
8877_A0A2X0R1Q7, AMHIJAGA_01426, DZANK-type domain-containing protein	9
6033_A2RKR3, leuC, 3-isopropylmalate dehydratase large subunit	8
9656_A0A5D4GGJ8, FYK05_06720, Uncharacterized protein	8
9807_A0A552Z8H2, FNJ53_01980, Alpha-mannosidase	7
10281_A0A0V8BSM9, LKF24_1801, Alpha-12-mannosidase	6
3378_A0A552YLX2, FNJ58_11860, Oligopeptide ABC transporter substrate-binding protein	6
9876_A0A418ZNE6, D4M07_01460, Uncharacterized protein	6
3891_A0A199YQC2, V425_08775, Pyruvate oxidase	6
7817_A0A1V0NGV3, FYK05_03555, Alpha-1,2-mannosidase	5
1952_A0A0V8F5Q1, KF282_0779, Oligopeptide ABC transporter periplasmic oligopeptide-binding protein OppA	5
	...
13553_R9QMT9, BM13_07, Putative regulatory protein	3
12251_A0A1V0PDI7, xseA, Exodeoxyribonuclease 7 large subunit	3
7936_Q8LTK0, orf45, Putative tail-host specificity protein	3
13543_A0A1E7G6X2, AJ89_02180, Rep_Org_C domain-containing protein	3
13611_A0A3S3LMM5, EO246_11575, Cyclase family protein	3
13991_Q9AZL2, orf53, Host-specificity	3
9863_A0A1V0NDZ0, LL275_0522, Prophage TAL protein	2
8742_A0A5D4GBJ0, FYK05_06745, PBECR4 domain-containing protein	2
2390_A2RJY0, purH, Bifunctional purine biosynthesis protein PurH	2
15711_T2DJZ9, ORF4, Putative tip pilin	2
15241_no_reference_sequence, nan, hypothetical protein	2
15249_A0A4Q7DS20, EQJ87_08675, Flippase	2
15541_no_reference_sequence, nan, hypothetical protein	2
15382_A0A1V0NRS4, LLJM4_pE38, Oligopeptide-binding protein oppA2	2
14262_S6ESE5, PphaDRAFT_1720, Uncharacterized protein	1
10831_A0A2X0R3D5, sle1, N-acetylmuramoyl-L-alanine amidase sle1	1
19150_A0A5C8JJ07, FVP42_09395, Site-specific integrase	1
13992_G6FCN2, LLCRE1631_01275, Uncharacterized protein	1
17262_no_reference_sequence, nan, hypothetical protein	1
17325_A0A5E9JFK6, BU174_10220, Pyruvate oxidase	1
6311_A0A2Z5Z9V4, mobD, MobD	1
11259_A0A5D4GEK9, FYK05_04250, Prophage_tail domain-containing protein	1
17344_A0A5E9JFK6, BU174_10220, Pyruvate oxidase	1
20154_A0A1K2HFG3, SAMN02746068_01581, Sortase A	1
4240_G8P3H7, leuC, 3-isopropylmalate dehydratase large subunit	1
8294_G9BNK8, nan, Hypothetical truncated plasmid mobilization protein D	1
19330_Q38319, nan, Uncharacterized protein	1
19603_no_reference_sequence, nan, hypothetical protein	1
17272_Q8LTK0, orf45, Putative tail-host specificity protein	1
7933_A2RMW7, ps457, Hypothetical serine/threonine-rich protein P11E10.02c in chromosome I	1

CCTGGCCA / TGGCCAGG

1266_A0A5D4G8C0, FYK05_07765, Aldo/keto reductase	230
1172_Q9CFG0, purH, Bifunctional purine biosynthesis protein PurH	214
1689_A0A3B0GSN0, D8M10_10975, Beta-galactosidase	197
1782_A0A2Z3KMV2, FYK05_01170, Uncharacterized protein	183
710_A0A2A9IHU5, BW151_00910, Ferrous iron transport protein B	181
123_A0A0A7T473, D4M07_02195, ABC transporter permease	173
4130_A0A4U1N5Z5, E6O52_02045, Uncharacterized protein	50
4322_Q9AZX9, orf50, Orf50	48
372_H5SVZ7, ychF, Ribosome-binding ATPase YchF	45
257_Q9CDT1, uppS, Isoprenyl transferase	44
2289_A0A552XE81, FNJ55_10690, Biotin--[acetyl-CoA-carboxylase] ligase	34
5234_A0A084A793, U725_02694, Serine protease	34
6046_A0A552YRJ9, FNJ58_08500, Beta-galactosidase	26
3918_A0A0H1RIT8, LKF67_1881, GntR family transcriptional regulator	26
6098_A0A3N6KPU0, D6118_12180, Phage tail protein	20
5929_A0A5D4GPE8, FYK05_00510, Phage tail protein	20
7777_A0A0V8E0U6, LMG9449_0698, Uncharacterized protein	14
815_A0A1V0ND63, FYK05_11130, Glycosyltransferase	11
8136_A0A2A9IC12, BW151_00480, Uncharacterized protein	10
2376_T0UJF2, LLT7_04785, Histidine kinase	10

6744_A0A1B1H1Y7, spaCB, Extracellular pilin protein, LPDTG anchored	8
8910_A0A199YQN0, V425_11060, Uncharacterized protein	8
7751_A0A3N6KPU0, D6118_12180, Phage tail protein	6
11756_A0A552XMU7, FNJ55_01905, Phage tail protein	5
11338_A0A5D4GE84, FYK05_04255, Phage tail protein	5
1658_A0A4U1N2C5, E6O52_09340, ABC transporter ATP-binding protein	4
9252_A0A552YL06, FNJ58_11545, Phage tail protein	4
13279_A0A3N6KPU0, D6118_12180, Phage tail protein	3
3169_T0VVJ5, LLT1_03515, Ig-like domain-containing protein	3
11304_S6FHF7, O9U_01445, Uncharacterized protein	3
14917_A0A5D4GPE8, FYK05_00510, Phage tail protein	2
285_O32810, deoD, Purine nucleoside phosphorylase DeoD-type	2
16119_A0A4U1N0F7, E6O52_07945, Beta-galactosidase	2
14336_no_reference_sequence, nan, hypothetical protein	2
11974_A0A0V8BWM0, D4M07_03645, Alpha-glucosidase	2
1794_G6FFL0, LLCRE1631_02303, Uncharacterized protein	2
15014_A0A0V8DLC1, LMG9449_2595, Uncharacterized protein	2
15591_A0A2A9IM14, BW154_06955, Mobilization protein	2
18325_A0A5D4GPE8, FYK05_00510, Phage tail protein	1
18303_A0A5D4GPE8, FYK05_00510, Phage tail protein	1
13281_A0A552YFD4, FNJ58_14740, Phage tail protein (Fragment)	1
10311_A0A2X0RBJ8, feoB, Ferrous iron transport protein B	1
12954_A0A0V8BMM8, LKF24_2067, Phage replicative DNA helicase repA	1
9754_A0A1V0NC18, LL275_pB20, LysM domain-containing protein	1
10596_A0A552YL06, FNJ58_11545, Phage tail protein	1
5641_A0A5E9JG24, BU174_09215, Phage holin	1
12816_A0A552XMU7, FNJ55_01905, Phage tail protein	1
18917_no_reference_sequence, nan, hypothetical protein	1
1129_A0A0V8BHN8, LKF24_2375, Copper-translocating P-type ATPase	1
18345_A0A5D4GPE8, FYK05_00510, Phage tail protein	1
841_H5SW69, ybcH, Hydrolase_4 domain-containing protein	1
18281_A0A5D4GPE8, FYK05_00510, Phage tail protein	1
19406_A0A2A9IPS2, BW154_00710, Glyco_trans_2-like domain-containing protein	1
12024_no_reference_sequence, nan, hypothetical protein	1
758_P23532, lacF, PTS system lactose-specific EIIA component	1
6237_A0A2Z3KDS5, LL14B4_04855, Phage holin	1
9815_A0A0V8DN08, LMG8520_0053, Beta-galactosidase	1
18227_A0A552YL06, FNJ58_11545, Phage tail protein	1

CGTATACG / CGTATACG

1963_A0A2X0PDC6, prtP, PIII-type proteinase	142
3921_A0A2Z5Z4J7, LLCC_0830, Polysaccharide biosynthesis export protein	54
4123_A0A1V0PCK8, LLJM3_2354, Polysaccharide biosynthesis export protein (Flippase)	40
4796_A0A1V0NI99, LL275_1998, Prophage BPP	39
1898_A0A1V0NSX1, BU174_02235, Transcriptional regulator	34
5391_T2F5Z1, kw2_1355, Cell surface protein	31
5452_A0A0B8QX55, JCM5805K_2776, Glycosyltransferase	31
4672_G6FFV4, LLCRE1631_02397, Uncharacterized protein	22
563_Q9CJD1, trpS, Tryptophan--tRNA ligase	19
1769_A0A1V0P5E4, LLUC06_2399, GRAM_POS_ANCHORING domain-containing protein	18
7718_A0A0V8AUD0, E34_0207, Alpha-D-GlcNAc alpha-12-L-rhamnosyltransferase	11
8178_G6FFV4, LLCRE1631_02397, Uncharacterized protein	10
8830_A0A0B8QN26, JCM5805K_1084, Nucleoside-diphosphate-sugar epimerases	10
6424_A0A5E9JFA6, BU174_10365, Polysaccharide transporter	9
8906_A0A2A9HLX0, BW151_11665, Mucus-binding protein	8
8849_A0A4S2TAU7, E5555_06535, Uncharacterized protein	7
9899_A0A3N6KLP2, D6118_13445, LPXTG cell wall anchor domain-containing protein	6
11423_A0A2X0R2U4, fokIM, Site-specific DNA-methyltransferase (adenine-specific)	5
12508_A0A2X0PDB6, AMHIJAGA_00674, Uncharacterized protein	4
12148_GCF_002078855.1_ASM207885v1_genomic, nan, hypothetical protein	3
13736_A0A5E9J9X4, BU174_12380, DNA-binding response regulator	3
12176_A0A1V0PCZ1, LLJM1_MP0022, DNA polymerase IV	3
15814_Q9KJ09, nan, Uncharacterized protein	2
15111_no_reference_sequence, nan, hypothetical protein	2
15140_no_reference_sequence, nan, hypothetical protein	2
4544_A0A1V0P5E4, LLUC06_2399, GRAM_POS_ANCHORING domain-containing protein	2
14359_A0A3Q9T941, nan, Acyltransferase	2
15711_T2DJZ9, ORF4, Putative tip pilin	2
12934_P16271, prtP, PI-type proteinase	1
16392_A0A2X0PDC6, prtP, PIII-type proteinase	1
20318_no_reference_sequence, nan, hypothetical protein	1
18841_no_reference_sequence, nan, hypothetical protein	1
21146_T2F5Z1, kw2_1355, Cell surface protein	1
22717_Q9AIQ2, prtP, PrtP	1
20500_A0A3N6KKI2, D6118_13535, DNA cytosine methyltransferase	1
6744_A0A1B1H1Y7, spaCB, Extracellular pilin protein, LPDTG anchored	1
6145_A0A3N6L7S0, AMHIJAGA_01412, Site-specific integrase	1
21145_T2F5Z1, kw2_1355, Cell surface protein	1
22691_A0A0M2ZP19, VN96_2651, Type VII secretion-associated serine protease mycosin, mycP	1
14064_T0WSZ7, LLT3_01170, Peptidase S8	1
20570_no_reference_sequence, nan, hypothetical protein	1
4660_A0A1V0NJL3, LLUC11_pB10, Uncharacterized protein	1
17754_G6FFV4, LLCRE1631_02397, Uncharacterized protein	1
18873_A0A2X0QYV8, AMHIJAGA_01004, Uncharacterized protein	1
13269_G6FFV4, LLCRE1631_02397, Uncharacterized protein	1
2694_A0A084ADJ4, U725_00506, Uncharacterized protein	1
9106_A0A4R5N401, C5L16_000978, GRAM_POS_ANCHORING domain-containing protein	1

CCGGGTAG / CTACCCGG

1578_Q9CG28, murI, Glutamate racemase	220
1963_A0A2X0PDC6, prtP, PIII-type proteinase	141
2048_A0A0A7T3I8, D4M07_07465, O-acetyl-L-homoserine sulfhydrolase / O-acetyl-L-serine sulfhydrolase	125
1913_A0A4V5Q0N0, E6O52_06855, O-acetylhomoserine aminocarboxypropyltransferase/cysteine synthase	88
6647_G8P8Z4, llh_14185, ADP-ribose pyrophosphatase	20
2145_A0A552XCE3, FNJ55_12305, Sugar ABC transporter permease	12
8619_A0A084A9S6, U725_01810, Ser/Thr protein Kinase	11
8098_Q9CG28, murI, Glutamate racemase	10
6868_A0A0V8AUQ3, CYU10_001094, O-acetyl-L-homoserine sulfhydrolase / O-acetyl-L-serine sulfhydrolase	9
9899_A0A3N6KLP2, D6118_13445, LPXTG cell wall anchor domain-containing protein	6
10450_G8P8Z4, llh_14185, ADP-ribose pyrophosphatase	6
1543_A0A1V0NCX9, LL275_0137, ABC transporter permease protein	3
9106_A0A4R5N401, C5L16_000978, GRAM_POS_ANCHORING domain-containing protein	3
12148_GCF_002078855.1_ASM207885v1_genomic, nan, hypothetical protein	3
11021_Q8LTK0, orf45, Putative tail-host specificity protein	3
15349_A0A098CZN7, rmlA1_2, Glucose-1-phosphate thymidylyltransferase 1	2
12934_P16271, prtP, PI-type proteinase	2
14064_T0WSZ7, LLT3_01170, Peptidase S8	2
15258_A0A224X4G4, RsY01_1176, N6_N4_Mtase domain-containing protein	2
19357_no_reference_sequence, nan, hypothetical protein	1
14203_G8P8Z4, llh_14185, ADP-ribose pyrophosphatase	1
12945_A0A1V0NN90, LLUC11_1145, Uncharacterized protein	1
18462_Q6QPZ1, llaJIM2, M2.LlaJI	1
14754_GCF_002148215.1_ASM214821v1_genomic, nan, hypothetical protein	1
16396_Q7M177, nan, Lactocepin (Fragments)	1
6213_A0A552XBB2, FNJ55_13240, DUF389 domain-containing protein	1
14082_A0A4Q7DLN6, EQJ87_11380, Uncharacterized protein	1
16392_A0A2X0PDC6, prtP, PIII-type proteinase	1

CCCGGCCC / GGGCCGGG

108_G8P4K5, hflX, GTPase HflX	186
536_A0A0V8F5M9, carA, Carbamoyl-phosphate synthase small chain	112
3062_G8P4E4, llh_1055, 6-aminohexanoate-cyclic-dimer hydrolase	93
4010_A0A0V8ERS3, fetB, Iron export ABC transporter permease subunit FetB	58
5352_A0A0D6E0F4, LACPI_2377, YbbM family protein	35
516_Q9CGY0, yjiF, Putative gluconeogenesis factor	33
696_A0A161VFB9, BU174_05970, Acetolactate synthase	24
7649_A0A1V0NZ40, LLUC06_0159, ABC superfamily ATP binding cassette transport system	13
7298_A0A0D6E0F4, LACPI_2377, YbbM family protein	13
2802_A0A552YTB2, xylB, Xylulose kinase	2
20087_A0A199YUE2, V425_04245, AAA_5 domain-containing protein	1
22765_A0A0D6E0F4, LACPI_2377, YbbM family protein	1
21673_G8P8X6, xylB, Xylulose kinase	1
21674_G8P8X6, xylB, Xylulose kinase	1
14761_GCF_002148215.1_ASM214821v1_genomic, nan, hypothetical protein	1
13026_A0A2Z3KEJ8, LL14B4_07710, Glycosyltransferase family 2 protein	1
21676_G8P8X6, xylB, Xylulose kinase	1

GCCGGGTA / TACCCGGC

1963_A0A2X0PDC6, prtP, PIII-type proteinase	141
2050_A0A0V8DZ16, FYK05_05830, Flavocytochrome c	112
3263_T0UQW3, pcp, Pyrrolidone-carboxylate peptidase	92
4149_A0A552XPH1, spxB, Pyruvate oxidase	52
4240_G8P3H7, leuC, 3-isopropylmalate dehydratase large subunit	44
5378_G8P9N3, llh_11870, Pyruvate oxidase	33
5617_A0A3N6KJ95, D6118_13870, Uncharacterized protein	29
5592_O87765, pcp, Pyrrolidone-carboxylate peptidase	29
6033_A2RKR3, leuC, 3-isopropylmalate dehydratase large subunit	22
5077_A0A2Z5Z723, leuC, 3-isopropylmalate dehydratase large subunit	20
6058_T0UQW3, pcp, Pyrrolidone-carboxylate peptidase	18
7270_O87765, pcp, Pyrrolidone-carboxylate peptidase	13
8598_WP_063283344, nan, hypothetical protein	11
9071_T0UQW3, pcp, Pyrrolidone-carboxylate peptidase	10
9222_GCF_002078375.2_ASM207837v2_genomic, nan, hypothetical protein	8
10237_A0A4R5MVY9, C5L16_001694, Aconitase domain-containing protein	7
3891_A0A199YQC2, V425_08775, Pyruvate oxidase	6
10335_T0UQW3, pcp, Pyrrolidone-carboxylate peptidase	6
9899_A0A3N6KLP2, D6118_13445, LPXTG cell wall anchor domain-containing protein	6
3097_T0UPL0, LLT1_10045, Uncharacterized protein	6
11422_no_reference_sequence, nan, hypothetical protein	5
11348_A0A2N5WAM8, CYU10_000147, Uncharacterized protein	5
2848_A0A1V0PIB8, BU174_08340, Competence protein CoiA	4
4606_Q9AZL2, orf53, Host-specificity	3
9106_A0A4R5N401, C5L16_000978, GRAM_POS_ANCHORING domain-containing protein	3
12934_P16271, prtP, PI-type proteinase	2
14064_T0WSZ7, LLT3_01170, Peptidase S8	2
12945_A0A1V0NN90, LLUC11_1145, Uncharacterized protein	1
17354_G8P9N3, llh_11870, Pyruvate oxidase	1
16392_A0A2X0PDC6, prtP, PIII-type proteinase	1
16396_Q7M177, nan, Lactocepin (Fragments)	1
14082_A0A4Q7DLN6, EQJ87_11380, Uncharacterized protein	1
19591_A5GYN0, nan, Putative antireceptor	1
11850_A0A552YZQ8, FNJ53_10515, Phage major capsid protein	1

CCCGCGGG / CCCGCGGG

1879_A0A199YU45, V425_04615, Uncharacterized protein	170
3735_A0A1V0P1M8, LLUC06_1164, Uncharacterized protein	46
1573_A0A1V0NDH8, FYK05_07950, Uncharacterized protein	46

3205_T0W656, LLT1_01675, Glyco_trans_2-like domain-containing protein	32
5642_T0VBR3, LLT3_02570, Uncharacterized protein	2
19492_A0A552YP05, FNJ58_09700, Transcription elongation factor GreAB	1

AGACCGGG / CCCGGTCT

1704_G6FA63, LLCRE1631_00406, VWFA domain-containing protein	167
1543_A0A1V0NCX9, LL275_0137, ABC transporter permease protein	148
1963_A0A2X0PDC6, prtP, PIII-type proteinase	138
2782_A0A1V0NTV8, BU174_07100, Lactoylglutathione lyase	112
367_Q9CIJ4, nadK, NAD kinase	111
2559_A0A1E7G3N0, AJ89_08730, Endonuclease	78
3425_T0TIN0, ilvA, L-threonine dehydratase	63
3931_A2RMB5, glgA, Glycogen synthase	57
710_A0A2A9IHU5, BW151_00910, Ferrous iron transport protein B	56
4759_T0TIN0, ilvA, L-threonine dehydratase	38
522_A0A3N6LTR1, carB, Carbamoyl-phosphate synthase large chain	34
5536_A0A5E9JLN0, glgA, Glycogen synthase	24
6459_T0UEH4, glgA, Glycogen synthase	18
72_U6EJD3, BN927_01516, Zinc ABC transporter, ATP-binding protein ZnuC	16
6554_A0A4U1N091, E6O52_07630, VWA domain-containing protein	15
4246_G6FA63, LLCRE1631_00406, VWFA domain-containing protein	15
9560_A0A418ZNM5, D4M07_01465, MPTase-PolyVal domain-containing protein	6
9899_A0A3N6KLP2, D6118_13445, LPXTG cell wall anchor domain-containing protein	6
9917_WP_014608333, nan, Putative glycosyltransferase EpsH	6
6853_A0A4U1N1K1, E6O52_07380, Phage repressor protein/antirepressor Ant	4
13504_A0A2X0R7W7, AMHIJAGA_01255, Uncharacterized protein	3
9106_A0A4R5N401, C5L16_000978, GRAM_POS_ANCHORING domain-containing protein	3
11776_G8P6W8, llh_9465, Glycogen synthase, ADP-glucose transglucosylase	3
13632_H5SZ75, hsdS, Probable specificity determinant HsdS	3
5065_A0A1V0NHS6, LL275_1874, Excinuclease ABC subunit B	2
15170_A0A3Q9TE96, nan, Glycosyltransferase	2
15187_no_reference_sequence, nan, hypothetical protein	2
12934_P16271, prtP, PI-type proteinase	1
13928_Q02V83, LACR_E6, DNA or RNA helicase of superfamily II	1
21023_A0A3N6N2R6, D6118_05790, VWA domain-containing protein	1
4241_A0A1V0NTX8, LLJM4_0896, Putative extracellular protein	1
20558_A0A4Q7DNQ3, EQJ87_10995, Nucleotidyltransferase domain-containing protein	1
22691_A0A0M2ZP19, VN96_2651, Type VII secretion-associated serine protease mycosin, mycP	1
14064_T0WSZ7, LLT3_01170, Peptidase S8	1
12121_A0A0A7T0D7, KF282_2340, Uncharacterized protein	1
16392_A0A2X0PDC6, prtP, PIII-type proteinase	1

CGCGCCCC / GGGGCGCG

2384_T0S925, dapB, 4-hydroxy-tetrahydrodipicolinate reductase	112
2796_A0A1E7G784, AJ89_00320, Short-chain dehydrogenase	107
619_A0A3B0H6A7, D8M10_04720, PTS fructose transporter subunit IIC	105
1957_A0A552YY26, FNJ58_01005, Sensor histidine kinase KdpD	101
3809_A0A0V8BPS7, D4M07_03110, ABC transporter ATP-binding protein	59
465_A0A2A5SJG1, RU90_GL002050, RelA_SpoT domain-containing protein	54
4393_A0A2X0R081, kdpD, Sensor protein KdpD	51
243_Q9CE80, aspS, Aspartate--tRNA ligase	35
468_T2F3F8, kw2_0426, Sugar ABC transporter ATP-binding protein	34
5298_A0A552XLW0, FNJ55_03155, Beta-glucosidase	32
6009_A0A1E7G3I2, AJ89_07565, Uncharacterized protein	25
7467_A0A0M2ZVV2, VN96_1126, Beta-glucosidase	16
714_A0A0V8CDJ9, ilvD, Dihydroxy-acid dehydratase	10
9370_G6F913, LLCRE1631_00006, Histidine kinase	8
9153_G8P8X7, llh_5075, Aldose 1-epimerase	5
1554_Q9CHW2, acyP, Acylphosphatase	4
12354_A0A098CZ60, LGMT14_01629, Uncharacterized protein	4
5630_A0A552YWD6, FNJ53_13330, DUF2479 domain-containing protein	4
1763_G6FDB0, LLCRE1631_01503, Glycosyltransferase	4
11744_T2F4F0, kw2_0576, Type II restriction-modification system restriction subunit	2
15229_D2BMH1, ycbI, Glycosyltransferase, family 2	2
19302_no_reference_sequence, nan, hypothetical protein	1

CCGATCGA / TCGATCGG

642_A0A2Z5Z4F5, LLCC_0789, Acyltransferase/SGNH hydrolase	112
2955_A0A552XE72, FNJ55_10670, Amidase domain-containing protein	96
319_Q9CJ54, tgt, Queuine tRNA-ribosyltransferase	93
3258_A0A5E9JJ43, BU174_03185, Aconitate hydratase	79
3234_A0A2X0PNV8, AMHIJAGA_03052, Putative type I restriction enzymeP M protein	77
2410_T0WRY4, LLT3_09475, 7,8-dihydroneopterin aldolase	76
3622_G6FDG3, LLCRE1631_01556, MobC domain-containing protein	72
3477_A0A552YXH0, FNJ53_12870, Type I restriction endonuclease subunit R	71
3932_A0A5D4GHY6, mobC, Plasmid mobilization relaxosome protein MobC	59
4138_T0U9S2, LLT1_00685, Mobilization protein	54
4435_A0A3B0GSK7, D8M10_12840, Type I restriction-modification system subunit M	46
5054_A2RKX4, telC, Putative tellurium resistance protein	35
2516_T0UPD5, LLT1_10230, RNA methyltransferase	34
6192_Q9CHS1, hsdR, Type I restriction enzyme R Protein	23
6220_O52273, HsdM, Modification subunit	21
6265_A0A2N5W9N0, CYU10_002307, Phage nucleotide-binding protein	21
7408_I7KHJ2, BN193_04835, Type I restriction-modification system,DNA-methyltransferase subunit M	14
7156_T0W4I1, LLT7_13655, Type I restriction enzyme R protein	14
7442_G6FDG3, LLCRE1631_01556, MobC domain-containing protein	14
5841_A0A170MYL3, AB996_0770, Aconitate hydratase	14
8288_A0A5E9JJ43, BU174_03185, Aconitate hydratase	12

9369_A0A552XE72, FNJ55_10670, Amidase domain-containing protein 9
9856_A0A2A5RV88, RU87_GL000612, Bacterial mobilization protein (MobC) 7
2027_GCF_000014545.1_ASM1454v1_genomic, nan, hypothetical protein 7
8777_A0A0V8BY67, LKF24_1364, Positive transcriptional regulator MutR family 6
7822_A0A4U1MYS5, E6O52_11305, Peptidase M23 6
3846_A0A1V0NCP2, FYK05_10350, Prophage protein 6
10904_G6FDG3, LLCRE1631_01556, MobC domain-containing protein 6
5171_A0A0V8CFH3, D4M07_04935, Glycosyltransferase family 2 protein 5
3172_H2AM39, hsdS, Putative methylase S 5
...
3978_A0A5E9JDB7, BU174_12270, Restriction endonuclease subunit S (Fragment) 2
10791_A0A2Z5Z8L4, citB, Aconitate hydratase 2
13248_A0A1V0P0Y0, LLUC06_0915, Prophage protein 2
9854_A0A4V6PJG7, C5L16_002413, Methylase_S domain-containing protein 2
10240_G1FE34, hsdM, Type I restriction-modification system modification subunit 2
13176_G0WKQ4, hsdR, Type I restriction enzyme R protein 2
12697_G6FDH0, LLCRE1631_01563, Site-specific DNA-methyltransferase (Adenine-specific) 2
6632_A0A4U1N837, E6O52_00555, Restriction endonuclease subunit S 2
17045_Q9CHS1, hsdR, Type I restriction enzyme R Protein 1
10785_G0WJU4, hsdM, Type I R/M system methylation subunit 1
17035_Q9CHS1, hsdR, Type I restriction enzyme R Protein 1
6488_T0UC80, LLT7_14850, Uncharacterized protein 1
16668_Q38338, nan, Uncharacterized protein 1
17073_Q9CHS1, hsdR, Type I restriction enzyme R Protein 1
20090_A0A1V0NFY0, LL275_1227, Helicase conserved C-domain protein 1
5877_A0A552YVN4, FNJ53_13700, Uncharacterized protein 1
7479_T0TLF0, LLT6_13060, Uncharacterized protein 1
8361_A0A4V6PJG7, C5L16_002413, Methylase_S domain-containing protein 1
21110_A0A1V0PEU4, C5L16_002090, Surface antigen 1
20119_A0A552YSK0, FNJ58_06870, Acylphosphatase 1
11862_no_reference_sequence, nan, hypothetical protein 1
15920_A0A5D4GD10, FYK05_04650, Type I restriction-modification system subunit M 1
5764_I6TDF7, hsdS, Type I restriction-modification HsdS subunit 1
6224_Q9CFQ8, pi334, Prophage pi3 protein 34 1
17054_T0W4I1, LLT7_13655, Type I restriction enzyme R protein 1
9947_A0A2N5W9N0, CYU10_002307, Phage nucleotide-binding protein 1
7631_A0A552XBI5, FNJ55_13115, Restriction endonuclease subunit S 1
132_A0A0A7T4D2, tmk, Thymidylate kinase 1
7516_G0WKQ4, hsdR, Type I restriction enzyme R protein 1
20210_no_reference_sequence, nan, hypothetical protein 1

CGGCCCCG / CGGGGCCG
526_Q9CF36, mutS2, Endonuclease MutS2 112
4001_A0A2Z5Z3C0, LLCC_0429, Uncharacterized protein 43
4697_A0A552XPG4, FNJ55_00330, Pyridine nucleotide-disulfide oxidoreductase 31
5532_A0A5E9JHL7, BU174_05870, MFS transporter 24
3642_A0A1V0PI20, C5L16_001902, Permease of the major facilitator superfamily 10
3451_H5SXL1, scrK, Fructokinase 10
9431_A0A2Z5Z3C0, LLCC_0429, Uncharacterized protein 8
10330_A0A5E9JLI1, BU174_00750, Uncharacterized protein 7
10747_no_reference_sequence, nan, hypothetical protein 6
9625_A0A1V0PEM0, LMA_00053, Nucleoside-diphosphate-sugar epimerase 6
2880_T0V5M5, LLT1_13160, Oxidoreductase 4
11990_A0A1V0P3T2, LLUC06_1890, Prophage protein 4
3361_G8P9P5, llh_11930, Putative Dihydrolipoamide dehydrogenase, Mercuric ion reductase 3
18540_A0A084AC92, U725_00901, Putative ABC transporter 1

CGGCCCGA / TCGGGCCG
81_A0A0B8QK93, recG, ATP-dependent DNA helicase RecG 72
3526_A0A199YVL8, V425_02170, Uncharacterized protein 60
3790_A0A1E7G234, AJ89_10765, Metal ABC transporter ATPase 28
3463_A0A2X0R6I6, AMHIJAGA_02577, Uncharacterized protein 27
4631_A0A1V0NQY6, LLUC11_2165, Phage related anti-repressor protein 23
4180_A0A418ZIU7, D4M07_09185, Bro-N domain-containing protein 20
7050_A0A2Z3KG78, LL14B4_01070, Cell wall anchor protein 18
6325_A0A1E7G234, AJ89_10765, Metal ABC transporter ATPase 18
6567_A0A1V0NTG4, LLJM4_0606, Calcium-transporting ATPase 9
13441_A0A418ZND6, D4M07_01410, LPXTG cell wall anchor domain-containing protein 3
5835_A0A418ZIU7, D4M07_09185, Bro-N domain-containing protein 3
13497_no_reference_sequence, nan, hypothetical protein 3
13864_A0A1E7G234, AJ89_10765, Metal ABC transporter ATPase 3
10276_T0UBL6, LLT1_04615, Cation_ATPase_N domain-containing protein 2
11638_A0A1V0NQY6, LLUC11_2165, Phage related anti-repressor protein 2
18572_A0A2X0PSS3, AMHIJAGA_01014, Uncharacterized protein 1
1918_A0A552YVJ0, FNJ58_03980, Cell wall anchor protein 1
6789_A0A1V0NJK7, LLUC11_pB17, Thiol reductase thioredoxin 1
4467_A0A3B0G751, D8M10_01580, Cell wall anchor protein 1
8339_A0A1V0P0V4, LLUC06_0885, Uncharacterized protein 1
831_A0A166YF72, llrD, DNA-binding response regulator 1

GCCGTGCC / GGCACGGC
306_H5SW22, pdhD, Dihydrolipoyl dehydrogenase 230
1590_A0A0V8C435, D4M07_00485, Glycosyltransferase 220
34_A0A5E9JXM6, nadE, NH(3)-dependent NAD(+) synthetase 175
2052_T0VLT5, LLT1_02685, MFS transporter permease 137
48_A0A0A7T7M6, FYK05_13175, Fructose-2,6-bisphosphatase 99
3957_A0A1B1RSL6, FNJ55_13995, ATP-dependent Clp protease ATP-binding subunit 64
1775_A0A0V8BZ72, LKF67_0660, Putative Lysophospholipase Monoglyceride lipase 63

334_P37282, groL, 60 kDa chaperonin	34
5617_A0A3N6KJ95, D6118_13870, Uncharacterized protein	29
5026_A0A1P8BLY2, DS98103_42, Major capsid protein	24
3489_A0A1P8BLY2, DS98103_42, Major capsid protein	19
6263_A0A1V0PDI6, LLJM1_MP0166, Type I restriction-modification system, HsdM subunit	19
7501_A0A023UB80, W34_003, Glycosyltransferase	14
8562_A0A3N6MT82, D6118_11635, DUF1642 domain-containing protein	11
8073_A0A0H1RNI6, VN91_1332, Poly-beta-1,6 N-acetyl-D-glucosamine synthase, pgaC	10
8821_A0A5E9JKG6, BU174_05125, Uncharacterized protein	10
8497_S6FJY9, lysS, Lysine--tRNA ligase	7
443_A9QSL8, gatB, Aspartyl/glutamyl-tRNA(Asn/Gln) amidotransferase subunit B	6
4553_A2RMX0, ps453, Phage tail component	5
10905_A0A1P8BLY2, DS98103_42, Major capsid protein	5
11499_A0A418ZNW2, D4M07_01400, Uncharacterized protein	5
714_A0A0V8CDJ9, ilvD, Dihydroxy-acid dehydratase	4
8060_A0A4S2T6Y6, E5555_05605, Uncharacterized protein	4
11809_T0VLT5, LLT1_02685, MFS transporter permease	4
12578_A0A084A8T2, U725_02153, Uncharacterized protein	4
11202_A0A4S2T788, E5555_06015, Uncharacterized protein	4
239_Q02WP2, rpoZ, DNA-directed RNA polymerase subunit omega	4
12176_A0A1V0PCZ1, LLJM1_MP0022, DNA polymerase IV	3
13440_A0A5D4GDQ0, FYK05_06695, Uncharacterized protein	3
11182_G8P518, llh_7820, ImpB/MucB/SamB family protein	3
10916_A0A2Z5ZA93, LLCC_2854, Major facilitator superfamily permease	3
3684_A0A1V0NR72, LLJM4_pA07, DNA polymerase IV	2
1991_A0A0M2ZS42, VN96_1520, EpsL	2
4614_A0A0M2ZS42, VN96_1520, EpsL	2
15509_A0A418ZNW2, D4M07_01400, Uncharacterized protein	2
19603_no_reference_sequence, nan, hypothetical protein	1
19344_Q9AYX4, nan, Uncharacterized protein	1
862_A0A1V0NYY6, LLUC06_0111, 1,4-alpha-glucan branching enzyme	1
6349_A0A1V0P5U2, LLJM3_pA46, DNA polymerase IV	1
10344_A0A3B0GFW2, D8M10_00945, Y-family DNA polymerase	1
19085_no_reference_sequence, nan, hypothetical protein	1
22184_A0A1P8BMH4, DS98204_41, Major capsid protein	1
19982_A0A1V0NHT1, LL275_1871, Prophage protein	1
8084_A0A0V8BEL0, LKF24_2652, Prophage protein	1
14239_A0A0B8QZG6, JCM5805K_1429, Nucleotidyltransferase/DNA polymerase	1
3623_A0A1V0NWM9, umuC, DNA polymerase IV	1
14077_A0A1V0NEY6, LL275_0837, Uncharacterized protein	1
19505_S6ER13, umuC, ImpB/MucB/SamB family protein	1

CGGTCCGA / TCGGACCG

2052_T0VLT5, LLT1_02685, MFS transporter permease	137
373_Q9CDW3, rplD, 50S ribosomal protein L4	112
2347_T2F4G9, kw2_0399, PSP1 C-terminal domain-containing protein	112
2187_T0UKY1, LLT1_12200, Prephenate dehydrogenase	112
831_A0A166YF72, llrD, DNA-binding response regulator	107
83_A0A0V8F6M6, FYK05_08005, RNA-binding transcriptional accessory protein	78
122_G6FDX0, ddl, D-alanine--D-alanine ligase	78
3230_T2F6V6, kw2_1688, LD-carboxypeptidase	38
6658_A0A418ZIX5, D4M07_09195, XRE family transcriptional regulator	20
1381_G6FCF2, LLCRE1631_01195, Muramoyltetrapeptide carboxypeptidase	17
81_A0A0B8QK93, recG, ATP-dependent DNA helicase RecG	15
7182_G8P7M9, llh_3910, Muramoyltetrapeptide carboxypeptidase	15
7681_A0A1V0NZ66, LLUC06_0274, Lipopolysaccharide biosynthesis protein	11
9653_A0A5D4GB36, FYK05_06705, LPXTG cell wall anchor domain-containing protein	8
8476_T2F698, kw2_1293, Transcriptional regulator GntR family	8
8856_D2BQT8, pksA, PKS acyl-CoA transferase	6
11809_T0VLT5, LLT1_02685, MFS transporter permease	4
677_A0A2X0SXC9, pspA, Phosphoserine phosphatase 1	3
11887_A0A0A7T1P6, D4M07_11905, AraC family transcriptional regulator	3
10916_A0A2Z5ZA93, LLCC_2854, Major facilitator superfamily permease	3
3095_A0A0A7SZ23, liaR, DNA-binding response regulator	2
14862_G8P7M9, llh_3910, Muramoyltetrapeptide carboxypeptidase	2
15064_A0A139MGA5, LACDD01_00380, FtsK domain-containing protein	2
14851_A0A5E9JI97, BU174_08255, LD-carboxypeptidase	2
17668_A0A2Z5Z5W9, LLCC_1312, Muramoyltetrapeptide carboxypeptidase	1
19489_A0A552YNZ2, FNJ58_09675, N-6 DNA methylase	1
14720_A0A1V0P2A0, LLUC06_1358, Integrase	1
18838_no_reference_sequence, nan, hypothetical protein	1
12980_A0A0A7T1P6, D4M07_11905, AraC family transcriptional regulator	1
16630_A0A0A7T1P6, D4M07_11905, AraC family transcriptional regulator	1
14744_A0A0V8ELJ8, LLUC06_1385, DNA methylase	1

GCGCGCCC / GGGCGCGC

286_T0UPZ4, murA, UDP-N-acetylglucosamine 1-carboxyvinyltransferase	186
669_A0A1E7G664, rny, Ribonuclease Y	112
2693_T0WRP3, mntH, Divalent metal cation transporter MntH	102
1737_T0UGJ2, LLT1_04820, Diguanylate cyclase	91
829_A0A2A9ICB9, BW151_00875, Uncharacterized protein	16
7540_A0A5E9JFJ5, BU174_11325, Cell division protein FtsK	15
11675_A0A5E9JKY2, mntH, Divalent metal cation transporter MntH	5
1784_A0A5D4GI34, FYK05_02365, Uncharacterized protein	4
5630_A0A552YWD6, FNJ53_13330, DUF2479 domain-containing protein	4
887_Q9CGL4, ykjB, Uncharacterized protein	2
13055_A0A0V8BFP4, LKF67_2540, Preprotein translocase secY subunit	1
11684_A0A0V8AQG8, E34_1368, Putative conjugal transfer protein	1

CCCACGGG / CCCGTGGG

1006_A0A0A7T596, D4M07_11450, DUF805 domain-containing protein	230
522_A0A3N6LTR1, carB, Carbamoyl-phosphate synthase large chain	226
1951_D2BKW8, agl, Alpha-amylase	130
3270_A0A0V8C5G6, LKF24_0728, Alpha-amylase	88
3575_T0VGS1, LLT3_04205, Endoglucanase	69
810_Q9CIX6, tmcAL, tRNA(Met) cytidine acetate ligase	17
6763_A0A3N6MZU3, D6118_10400, NAD-dependent epimerase/dehydratase family protein	12
3735_A0A1V0P1M8, LLUC06_1164, Uncharacterized protein	11
8086_A0A1P8BKE1, DS38502_54, Tail protein	9
11423_A0A2X0R2U4, fokIM, Site-specific DNA-methyltransferase (adenine-specific)	5
4795_A0A5D4G6Q9, FYK05_09300, Cof-type HAD-IIB family hydrolase	4
2362_T0S545, LLT6_04780, Pantothenic acid transporter pant	4
12456_A0A418ZKY6, D4M07_05935, Phage tail protein	4
5396_A0A552YVR1, FNJ58_02320, Uncharacterized protein	3
20345_A0A3D3NP33, DIS86_07410, ABC transporter ATP-binding protein	1
1335_A0A0A7T053, LL14B4_03000, ABC transporter permease	1
19722_A0A1B1IME2, DS50901_47, Putative distal tail protein	1
13051_A0A0V8C7W0, LKF24_1647, Maltose O-acetyltransferase	1
14727_A0A0V8BJE9, LLUC06_1366, Methyltransferase	1
19876_T0UQM4, LLT1_06740, HTH cro/C1-type domain-containing protein	1

GGGGCCCC / GGGGCCCC

1430_Q9CDR9, rliDA, Transcriptional regulator	151
2767_A0A0V8EUY9, FYK05_04215, Internalin	112
3928_A0A0V8EUY9, FYK05_04215, Internalin	62
2317_G8P392, llh_0200, Dihydrolipoamide acetyltransferase component of pyruvate dehydrogenase complex	34
473_A0A5D4GIP3, FYK05_02620, ATP-dependent Clp protease ATP-binding subunit	34
9044_A0A0V8AUN5, E34_0322, Glycerophosphodiester phosphodiesterase	4
6336_T0UK80, LLT1_02475, Uncharacterized protein	2
21687_A0A5D4G946, FYK05_08885, LacI family DNA-binding transcriptional regulator	1
21688_A0A2A5SKI8, RU90_GL000864, Catabolite control protein B	1
