# Supplementary material for: Machine learning predicts and provides insights into milk acidification rates of Lactococcus lactis
Source: PLoS One. 2021 Mar 15;16(3):e0246287. doi: 10.1371/journal.pone.0246287 (PMC7959382; doi:10.1371/journal.pone.0246287)
Supplement: S4 File — (PDF) [file pone.0246287.s008.pdf]

The four Pfam domains in PrtP and the genes in which they were present

```
PF00082.17
1963_A0A2X0PDC6, prtP, PIII-type proteinase                                              142
3540_A0A1V0P5Q8, LLCC_2838, Serine protease                                               72
4315_Q48674, nisP, NisP                                                                   23
9212_A0A2A5RPY4, RT41_GL000252, AAA ATPase                                                 8
10689_no_reference_sequence, nan, hypothetical protein                                     6
9636_A0A2A9HY28, BW151_06580, Subtilisin                                                   6
9899_A0A3N6KLP2, D6118_13445, LPXTG cell wall anchor domain-containing protein             6
13847_Q48674, nisP, NisP                                                                   3
9014_H5SXJ9, nisP, Cleave leader peptide/cell wall-associated serine proteasee proteinase  3
9106_A0A4R5N401, C5L16_000978, GRAM_POS_ANCHORING domain-containing protein                3
13846_A0A223DQK0, pLd8_4, Serine protease                                                  3
12934_P16271, prtP, PI-type proteinase                                                     2
14064_T0WSZ7, LLT3_01170, Peptidase S8                                                     2
16396_Q7M177, nan, Lactocepin (Fragments)                                                  2
16392_A0A2X0PDC6, prtP, PIII-type proteinase                                               1
21287_T0THE7, LLT6_12615, Peptidase_S8 domain-containing protein                           1
22693_A0A1V0PDD3, LLJM1_MP0161, Lactoceptin PrtP                                           1
22700_Q7M177, nan, Lactocepin (Fragments)                                                  1
22708_P16271, prtP, PI-type proteinase                                                     1
22699_Q7M177, nan, Lactocepin (Fragments)                                                  1
19732_no_reference_sequence, nan, hypothetical protein                                     1
16395_A0A2X0PDC6, prtP, PIII-type proteinase                                               1
21290_Q48674, nisP, NisP                                                                   1
21288_Q48674, nisP, NisP                                                                   1
21289_A0A1B1RSN4, nisP, Nisin leader peptide-processing serine protease                    1
13210_GCF_002078415.1_ASM207841v1_genomic, nan, hypothetical protein                       1
dtype: int64
```

```
PF02225.17
1963_A0A2X0PDC6, prtP, PIII-type proteinase                                  142
9899_A0A3N6KLP2, D6118_13445, LPXTG cell wall anchor domain-containing protein     6
9106_A0A4R5N401, C5L16_000978, GRAM_POS_ANCHORING domain-containing protein        3
16395_A0A2X0PDC6, prtP, PIII-type proteinase                                       1
22693_A0A1V0PDD3, LLJM1_MP0161, Lactoceptin PrtP                                   1
22708_P16271, prtP, PI-type proteinase                                             1
16392_A0A2X0PDC6, prtP, PIII-type proteinase                                       1
14064_T0WSZ7, LLT3_01170, Peptidase S8                                             1
dtype: int64
```

```
PF06280.7
1963_A0A2X0PDC6, prtP, PIII-type proteinase                                  142
9899_A0A3N6KLP2, D6118_13445, LPXTG cell wall anchor domain-containing protein     6
9106_A0A4R5N401, C5L16_000978, GRAM_POS_ANCHORING domain-containing protein        3
12934_P16271, prtP, PI-type proteinase                                             2
14064_T0WSZ7, LLT3_01170, Peptidase S8                                             2
16396_Q7M177, nan, Lactocepin (Fragments)                                          1
22694_P15293, prt, PII-type proteinase                                             1
16392_A0A2X0PDC6, prtP, PIII-type proteinase                                       1
dtype: int64
```

```
PF00746.16
1769_A0A1V0P5E4, LLUC06_2399, GRAM_POS_ANCHORING domain-containing protein                    168
1963_A0A2X0PDC6, prtP, PIII-type proteinase                                                   144
2020_A0A3N6MZK3, D6118_10070, LPXTG cell wall anchor domain-containing protein                126
2520_T0V5N0, LLT3_01765, Internalin                                                           110
2767_A0A0V8EUY9, FYK05_04215, Internalin                                                      104
2859_A0A1V0NK65, LLUC11_0135, GRAM_POS_ANCHORING domain-containing protein                     84
4247_A0A5E9JJV4, BU174_01535, GRAM_POS_ANCHORING domain-containing protein                     52
4544_A0A1V0P5E4, LLUC06_2399, GRAM_POS_ANCHORING domain-containing protein                     47
4944_A0A2Z5Z6B3, LACR_1517, GRAM_POS_ANCHORING domain-containing protein                       32
5080_T0VU01, LLT1_04715, Uncharacterized protein                                               30
3504_A0A2Z5Z6H7, LLT1_10265, Uncharacterized protein                                           24
6299_A0A4U1N3S2, E6052_05930, LPXTG cell wall anchor domain-containing protein (Fragment)      21
6963_A0A1B1H1Y6, spaA, Extracellular pilin protein, LPSTG anchored                             18
6713_A0A1V0PHK3, C5L16_001479, Cell surface protein                                            16
3732_Q9CG97, ymcF, Uncharacterized protein                                                     13
7574_T0VAA5, LLT3_04230, Gram_pos_anchor domain-containing protein                             12
3928_A0A0V8EUY9, FYK05_04215, Internalin                                                       12
3202_A0A1V0NRX8, LLJM4_0127, Uncharacterized protein                                           11
8999_A0A084AAA5, U725_01392, Putative outer membrane protein (Fragment)                         10
8871_A0A552YWU7, FNJ58_01740, LPXTG cell wall anchor domain-containing protein                 10
8411_A2RKD6, llmg_1152, GRAM_POS_ANCHORING domain-containing protein                            8
8906_A0A2A9HLX0, BW151_11665, Mucus-binding protein                                             8
3989_A0A2Z5Z2W1, LLT1_06215, Uncharacterized protein                                            8
9899_A0A3N6KLP2, D6118_13445, LPXTG cell wall anchor domain-containing protein                  7
10300_T2DII5, muc, Mucus-binding protein                                                        6
10720_A0A2A9IQN2, BW154_09195, GRAM_POS_ANCHORING domain-containing protein                     6
10746_no_reference_sequence, nan, hypothetical protein                                          6
6708_T0UQ06, LLT1_06215, Uncharacterized protein                                                5
12703_A0A1V0NRX8, LLJM4_0127, Uncharacterized protein                                           4
4672_G6FFV4, LLCRE1631_02397, Uncharacterized protein                                           4
                                                                                              ...
1570_A0A1V0P0P3, LLUC06_0730, Uncharacterized protein                                           4
9106_A0A4R5N401, C5L16_000978, GRAM_POS_ANCHORING domain-containing protein                     4
```

13855_T0UV88, LLT1_10265, Gram_pos_anchor domain-containing protein	3
13919_A0A3D4RHC1, DIW25_04035, GRAM_POS_ANCHORING domain-containing protein	3
15529_A0A2A9IB35, BW151_00735, GRAM_POS_ANCHORING domain-containing protein	2
16392_A0A2X0PDC6, prtP, PIII-type proteinase	2
15710_T2DKF6, yhhB2, YhhB2	2
14064_T0WSZ7, LLT3_01170, Peptidase S8	2
20157_A0A1K2HGU6, SAMN02746068_01579, LPXTG-motif cell wall anchor domain-containing protein	1
20133_A0A3D3NPL6, DIS86_08420, Isopeptide-forming domain-containing fimbrial protein	1
20240_no_reference_sequence, nan, hypothetical protein	1
13269_G6FFV4, LLCRE1631_02397, Uncharacterized protein	1
20683_A0A084ABB5, U725_01233, GRAM_POS_ANCHORING domain-containing protein (Fragment)	1
12934_P16271, prtP, PI-type proteinase	1
22710_A0A2X0PDC6, prtP, PIII-type proteinase	1
20155_A0A2A5S6T5, RU88_GL001714, LPXTG-domain-containing protein cell wall anchor domain	1
11049_A0A3S3M7M3, EO246_00740, LPXTG cell wall anchor domain-containing protein	1
22707_Q49SG9, prtP, Lactocepin	1
21761_A0A1V0NBY0, LL275_pA088, Mucus-binding protein	1
17724_G6FFV5, LLCRE1631_02398, GRAM_POS_ANCHORING domain-containing protein	1
20896_A0A1V0NRX8, LLJM4_0127, Uncharacterized protein	1
22395_A0A098CZ61, sraP, Serine-rich adhesin for platelets	1
14553_H5SW98, ybeF, GRAM_POS_ANCHORING domain-containing protein	1
20894_A0A1V0NRX8, LLJM4_0127, Uncharacterized protein	1
22691_A0A0M2ZP19, VN96_2651, Type VII secretion-associated serine protease mycosin, mycP	1
17014_G6FEA7, LLCRE1631_01850, GRAM_POS_ANCHORING domain-containing protein	1
20897_A0A1V0P6E8, LLJM3_0145, Outer membrane protein	1
5834_A0A084A7Q8, U725_02535, Uncharacterized protein (Fragment)	1
17016_A0A3N6MZK3, D6118_10070, LPXTG cell wall anchor domain-containing protein	1
14077_A0A1V0NEY6, LL275_0837, Uncharacterized protein	1
