# Supplementary material for: Machine learning predicts and provides insights into milk acidification rates of Lactococcus lactis
Source: PLoS One. 2021 Mar 15;16(3):e0246287. doi: 10.1371/journal.pone.0246287 (PMC7959382; doi:10.1371/journal.pone.0246287)
Supplement: S5 File — (PDF) [file pone.0246287.s009.pdf]

Complete RefSeq genomes

GCF_000006865.1_ASM686v1_genomic.fa
GCF_000009425.1_ASM942v1_genomic.fa
GCF_000014545.1_ASM1454v1_genomic.fa
GCF_000025045.1_ASM2504v1_genomic.fa
GCF_000143205.1_ASM14320v1_genomic.fa
GCF_000192705.1_ASM19270v1_genomic.fa
GCF_000236475.1_ASM23647v1_genomic.fa
GCF_000312685.1_ASM31268v1_genomic.fa
GCF_000344575.1_ASM34457v1_genomic.fa
GCF_000468955.1_ASM46895v1_genomic.fa
GCF_000478255.1_ASM47825v2_genomic.fa
GCF_000479375.3_ASM47937v3_genomic.fa
GCF_000807375.1_ASM80737v1_genomic.fa
GCF_002078375.2_ASM207837v2_genomic.fa
GCF_002078415.1_ASM207841v1_genomic.fa
GCF_002078435.1_ASM207843v1_genomic.fa
GCF_002078475.2_ASM207847v2_genomic.fa
GCF_002078495.1_ASM207849v1_genomic.fa
GCF_002078615.2_ASM207861v2_genomic.fa
GCF_002078765.2_ASM207876v2_genomic.fa
GCF_002078855.1_ASM207885v1_genomic.fa
GCF_002078895.1_ASM207889v1_genomic.fa
GCF_002078915.1_ASM207891v1_genomic.fa
GCF_002078935.1_ASM207893v1_genomic.fa
GCF_002078955.1_ASM207895v1_genomic.fa
GCF_002078975.2_ASM207897v2_genomic.fa
GCF_002078995.2_ASM207899v2_genomic.fa
GCF_002148215.1_ASM214821v1_genomic.fa
GCF_002804185.1_ASM280418v1_genomic.fa
GCF_002804285.1_ASM280428v1_genomic.fa
GCF_002895225.1_ASM289522v1_genomic.fa
GCF_003176835.1_ASM317683v1_genomic.fa
GCF_003394085.1_ASM339408v1_genomic.fa
GCF_003966935.1_ASM396693v1_genomic.fa
GCF_900088425.1_A12_genomic.fa
