# Supplementary material for: Machine learning predicts and provides insights into milk acidification rates of Lactococcus lactis
Source: PLoS One. 2021 Mar 15;16(3):e0246287. doi: 10.1371/journal.pone.0246287 (PMC7959382; doi:10.1371/journal.pone.0246287)
Supplement: S11 File — Gene names and corresponding Uniprot IDs found in the strains. (PDF) [file pone.0246287.s015.pdf]

pH homeostasis genes not found in feature importance analysis

Table 1: **Genes for pH regulation in *L. lactis* which did not show up among the most important features in feature importance analysis.**

Gene	Strains	Uniprot IDs
F_1F_0-ATPase		
atpE	342	T0VYB2
atpB	342	H5T0Y3
atpF	342	P0A2Z1
atpH	342	Q93MY9, A2RMI5
atpA	342	A0A1E7G5Y3
atpG	342	Q9CER9, Q9RAU1
atpD	342	Q9CES0
atpC	342	Q9CES1
Glutamate decarboxylase		
gadC	322	A0A0V8BGA8, G6FB03, A0A1V0NV72, S6F093, G8P477, A0A0M2ZSP7, A0A1V0PHP7, A0A1V0PHH6
gadB	342	A0A0B8QP21, A0A2Z5Z7D0
gadR	330	A0A0H1RNV3, A0A3N6LC24
Arginine deiminase (ADI)		
arcA	337	P58013, Q9K576, A0A1V0NX69
arcB	342	P0C2U0
arcC	342	G3ACV8, A0A5D4FS98, G8P9K7, A0A1E7G1U6, A0A2Z5Z4Q5
arcD	291	A2RNI5, Q9CE19, G0WJT2, A0A2A5SRB6
Malolactic fermentation		
Malolactic enzyme	342	A0A0V8DT35, T0VDN6, A0A161U1P3, H5SYD7

For some genes multiple Uniprot IDs were found in the strains. Second column indicates how many strains contained at least one copy of the gene by one of the Uniprot IDs in third column. The genes could be present in more strains.
